# Silkworm excrement-derived carbon dots for sonodynamic bacterial killing and antioxidant–inflammatory modulation

**DOI:** 10.1039/d6ra05433a

**Published:** 2026-09-02

**Authors:** Yuxuan Ren, Wenhui Cao, Hui Wang, Jianing Zheng, Yongfang Wang, Anqi Liu, Chao Liu, Xin Pang, Zhenzhen Wang

**Affiliations:** a Academy of Chinese Medical Sciences, Henan University of Chinese Medicine Zhengzhou 450046 China wzlinxi@163.com; b Department of Rheumatology, The First Affiliated Hospital of Henan University of Chinese Medicine Zhengzhou 450046 China; c School of Pharmacy, Henan University of Chinese Medicine Zhengzhou 450046 China pangxin116@163.com

## Abstract

Persistent oxidative inflammation complicates the treatment of multidrug-resistant (MDR) bacterial infections. Sonodynamic therapy (SDT) enables ultrasound-activated bacterial killing but does not directly alleviate infection-associated oxidative stress. Here, phenylboronic acid (PBA)-functionalized carbon dots (PS-CDs) were prepared from silkworm excrement extract and PBA-terminated poly(ethylene glycol) (PBA-PEG-PBA) through a one-pot hydrothermal process. The PS-CDs showed spectroscopic features consistent with partially retained chlorophyll-derived chromophores, while PBA-PEG-derived groups improved aqueous dispersion and promoted bacterial association. Under ultrasound irradiation, PS-CDs generated reactive oxygen species (ROS) and reduced viable MDR *Pseudomonas aeruginosa* by 4.81 log_10_, accompanied by deoxyribonucleic acid and protein leakage. In the absence of ultrasound, PS-CDs scavenged 2,2′-azino-bis(3-ethylbenzothiazoline-6-sulfonic acid) radical cations (ABTS˙^+^), 2,2-diphenyl-1-picrylhydrazyl radicals (DPPH˙), and superoxide anions in concentration- and time-dependent manners. In lipopolysaccharide-stimulated RAW264.7 macrophages, PS-CDs reduced intracellular ROS and shifted CD86/CD206 expression toward an anti-inflammatory phenotype, with CD206-associated fluorescence accounting for 91.4% of the combined CD86/CD206 signal. Enzyme-linked immunosorbent assays further showed partial reductions in tumor necrosis factor-α, interleukin-6, and interleukin-1β secretion, together with increased transforming growth factor-β secretion. These findings provide an *in vitro* proof of concept for integrating ultrasound-activated bacterial killing with antioxidant and inflammatory modulation in a biomass-derived carbon dot platform.

## Abstract 2

Silkworm excrement-derived PS-CDs combine PBA-mediated bacterial association with ultrasound-activated ROS generation for MDR bacterial killing. Without ultrasound, intrinsic ROS scavenging supports antioxidant and inflammatory modulation.

## Graphical abstract

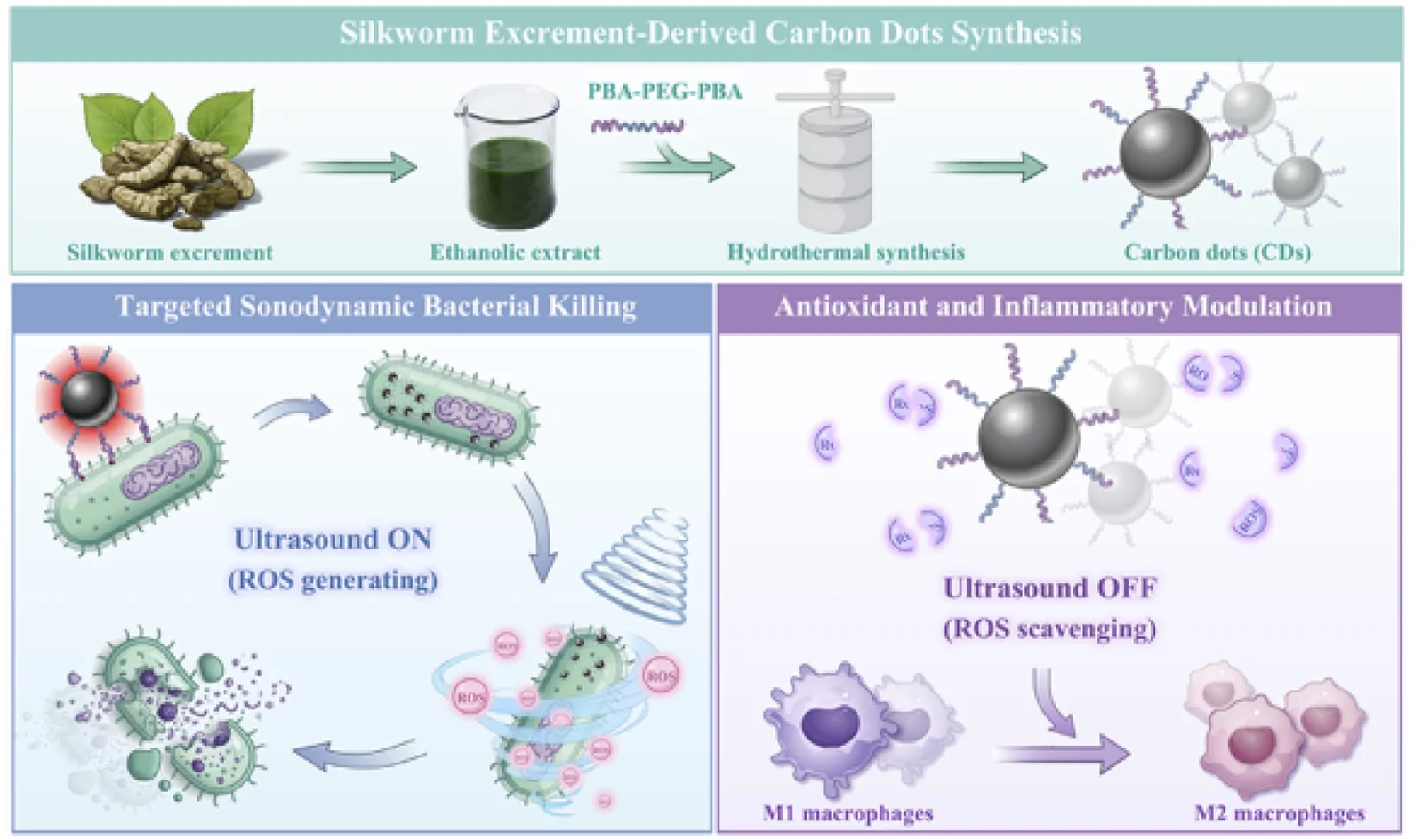

## 1. Introduction

The increasing prevalence of multidrug-resistant (MDR) bacterial infections poses a major challenge to conventional antibiotic therapy and has created an urgent need for alternative antibacterial strategies.^1,2^ However, bacterial elimination alone may not be sufficient for effective infection control. Persistent infections are often accompanied by excessive inflammation and elevated levels of endogenous reactive oxygen species (ROS), which disrupt local redox homeostasis and contribute to progressive tissue damage. In turn, sustained oxidative inflammation can delay tissue recovery and compromise host defense.^3–5^ Therefore, therapeutic strategies that combine bacterial killing with regulation of infection-associated oxidative stress may provide advantages over bactericidal treatment alone.

Sonodynamic therapy (SDT), which uses low-intensity ultrasound (US) to activate sonosensitizers for localized ROS generation, is an emerging antibacterial modality. Compared with light-based approaches, US offers greater tissue penetration and allows external control over the duration and location of sensitizer activation.^6,7^ The mechanisms underlying SDT remain incompletely understood and may involve acoustic cavitation, sonoluminescence, localized thermal effects, and sensitizer-mediated energy or electron transfer. In one proposed pathway, cavitation-associated sonoluminescence excites sensitizer chromophores, followed by energy or electron transfer to surrounding oxygen or water to generate ROS such as singlet oxygen (^1^O_2_) and hydroxyl radicals (˙OH). The contribution of each pathway depends on both the physicochemical properties of the sensitizer and the applied ultrasound conditions. Porphyrin and chlorophyll derivatives are widely investigated as sensitizers because of their extended π-conjugated structures.^8,9^ However, many conventional organic sensitizers are hydrophobic and have limited affinity for bacterial surfaces, which can restrict their dispersion and local interaction with pathogens.^10^ Moreover, their primary function is ROS generation. They do not inherently address the excessive oxidative stress that may persist in infected tissues after antibacterial treatment.^11,12^ Integrating ultrasound-triggered ROS generation with subsequent ROS scavenging may therefore provide a complementary approach to bacterial killing and oxidative stress regulation.

Carbon dots (CDs) are zero-dimensional carbonaceous nanomaterials with tunable surface chemistry and precursor-dependent optical and redox properties.^13,14^ Biomass-derived CDs can retain heteroatoms, surface functional groups, and chromophoric features originating from their precursors, although the extent of retention depends strongly on the carbonization conditions. Chlorophyll- and chlorophyllin-derived CDs have previously been reported to exhibit red fluorescence, energy or electron transfer, photodynamic activity, and other redox-related properties.^15^ Thus, the use of chlorophyll-related precursors and their associated ROS activity is not unique to the present study. Previous studies, however, have mainly employed purified chlorophyll or chlorophyllin and focused on fluorescence imaging, photodynamic therapy, or cancer-related applications. The combination of ultrasound-responsive antibacterial activity and intrinsic antioxidant regulation within a multicomponent biomass-derived CD system remains less explored.

Silkworm excrement (*Excrementum Bombycis*) is a naturally multicomponent biomass containing chlorophyll-derived pigments together with flavonoid and polyphenolic constituents.^16–18^ These components provide a potential chemical basis for combining sensitizer-associated chromophoric features with radical-scavenging activity. We hypothesized that controlled hydrothermal carbonization of silkworm excrement could preserve some chlorophyll-associated chromophoric features while generating oxygen-containing and phenolic surface groups capable of radical scavenging.^19,20^ Under US irradiation, the chromophoric components are expected to perform transient ROS generation for bacterial killing. After US cessation, ROS generation would terminate, while the intrinsic radical-scavenging activity of the CDs would remain available for subsequent redox regulation. This design therefore focuses on the functional integration of externally controlled sonodynamic antibacterial activity with antioxidant modulation rather than on precursor novelty alone.

**Fig. 1:**
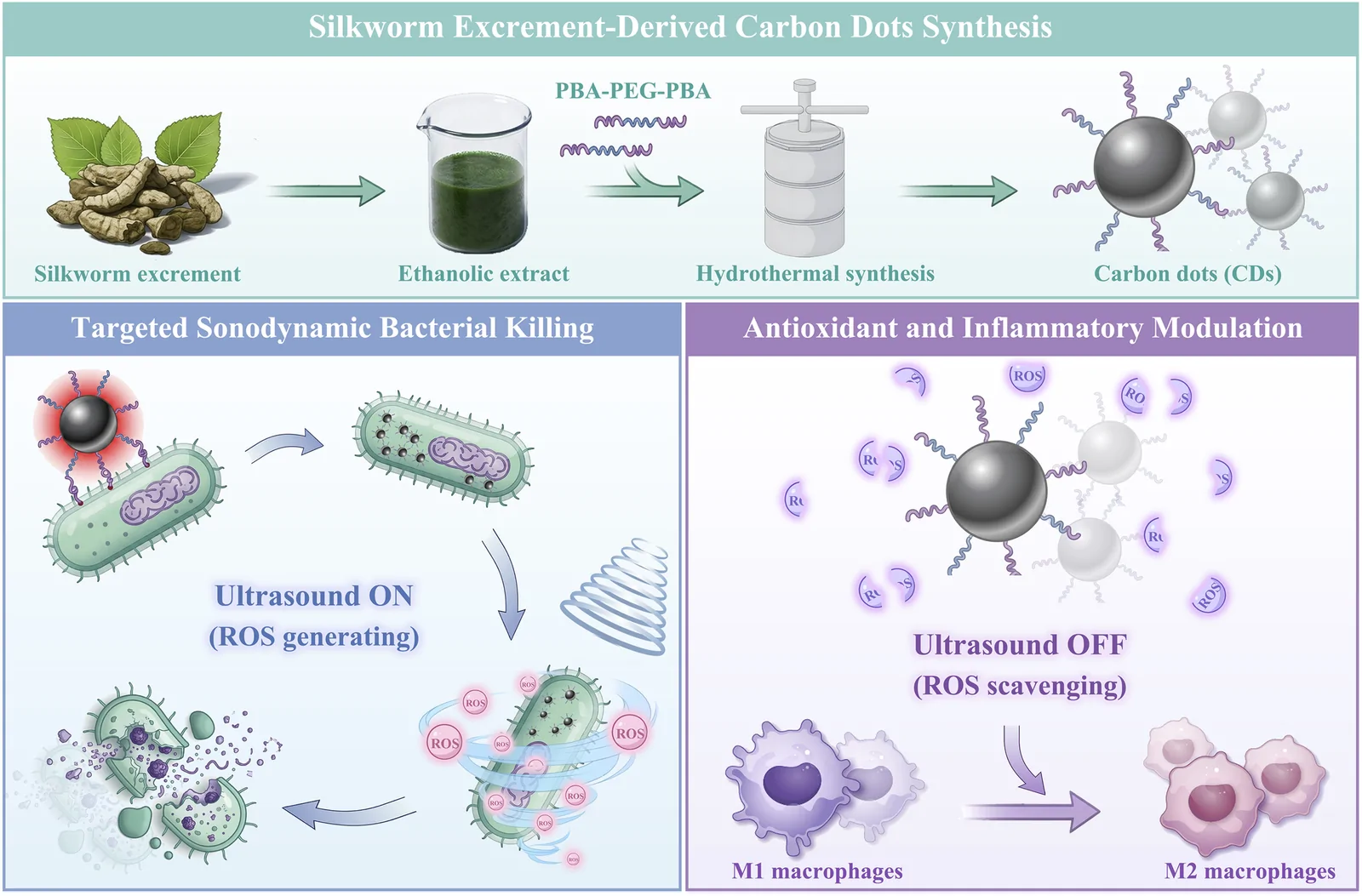
Schematic illustration of the preparation and proposed functions of silkworm excrement-derived CDs, including PBA-mediated bacterial association, ultrasound-triggered ROS generation for sonodynamic bacterial killing, and subsequent ROS scavenging for oxidative and inflammatory modulation.

Here, silkworm excrement extract and PBA-terminated poly(ethylene glycol) (PBA-PEG-PBA) were used as co-precursors to prepare PBA-containing CDs (PS-CDs) through a one-pot hydrothermal process (Fig. 1). PBA-PEG-derived surface groups were incorporated to improve aqueous dispersibility and promote bacterial association through interactions with *cis*-diol-containing surface structures. Spectroscopic characterization showed optical features consistent with partially retained chlorophyll-derived chromophores. Under US irradiation, PS-CDs generated ROS and reduced viable clinically isolated MDR *Pseudomonas aeruginosa* by 4.81 log_10_, accompanied by membrane damage and leakage of intracellular components. In the absence of US, PS-CDs scavenged multiple radical species and reduced intracellular ROS in lipopolysaccharide-stimulated RAW264.7 macrophages. They also partially decreased tumor necrosis factor-α, interleukin-6, and interleukin-1β secretion while increasing transforming growth factor-β secretion. These findings provide an *in vitro* proof of concept for integrating ultrasound-activated bacterial killing with antioxidant and inflammatory modulation in a biomass-derived CD platform.

## 2. Experimental section/methods

### 2.1. Reagents and materials

Silkworm excrement was purchased from Henan Zhangzhongjing Traditional Chinese Medicine Co., Ltd (Zhengzhou, China). Phenylboronic acid-polyethylene glycol 1000-phenylboronic acid (PBA-PEG1K-PBA) was purchased from Xi'an Ruixi Biotechnology Co., Ltd (Xi'an, China). 2,7-Dichlorodihydrouorescein diacetate (DCFH-DA) probe was purchased from Beyotime (Shanghai, China). Live/dead bacterial viability kits L7012 was purchased from Invitrogen (Carlsbad, CA, USA). Lipopolysaccharide (LPS) and other reagents were purchased from Shanghai Aladdin Biochemical Technology Co., Ltd (Shanghai, China). PBS buffer solution (10 mM) was purchased from Titan Scientific Co., Ltd (Shanghai, China). All chemicals listed above were used without any further purification. Ultrapure water (≥18 MΩ cm) was generated by a Hitachi water purification system (Tokyo, Japan) and used throughout the whole experiments.

### 2.2. Preparation of the PS-CDs

5 g of silkworm excrement was thoroughly ground in a mortar. Subsequently, it was mixed with 30 mL of ethanol in a beaker. The mixture was stirred continuously for 6 h in the dark, followed by ultrasonication in a water bath for 30 min to facilitate chlorophyll extraction.^21,22^ The chlorophyll extraction yield of the silkworm excrement extract, quantified by UV-vis spectroscopy, was 9.17 mg g^−1^ of raw silkworm excrement. After extraction, the mixture was filtered to remove the solid residue, and the resulting supernatant containing chlorophyll was transferred to a reactor with 2.5 g PBA-PEG1K-PBA for hydrothermal treatment at 150 °C for 6 h.^23,24^ Upon completion of the reaction, the solution was filtered again to purify it, and then placed into a dialysis bag with a molecular weight cutoff of 1000 Da for further refinement. Finally, ethanol was removed from the solution by rotary evaporation, and the product was resuspended in distilled water and stored at 4 °C until further use. After purification and lyophilization, the mass yield of PS-CDs was determined to be 9.09%.

### 2.3. Characterization of the PS-CDs

The morphology of the PS-CDs was examined using transmission electron microscopy (TEM, FEI Tecnai F20) operated at an acceleration voltage of 200 kV. The size distribution, polydispersity index (PDI), and zeta potential of the PS-CDs were determined using a Zetasizer (90Plus PALS, NanoBrook). UV-vis-NIR absorption spectra and fluorescence spectra were recorded using a microplate reader (SpectraMax iD5, Molecular Devices). The photoluminescence quantum yield (PLQY) of PS-CDs was measured using an FLS1000 fluorescence spectrometer (Edinburgh Instruments Ltd, Livingston, UK) with an excitation wavelength of 678 nm. Fourier transform infrared (FTIR) spectroscopy (Thermo Fisher Scientific, Waltham, MA, USA) was employed to obtain the spectra of silkworm excrement extract, PBA-PEG-PBA, and final PS-CDs in the wavenumber range of 500–4000 cm^−1^.

### 2.4. Determination of US-activated ROS generation

The US-activated ROS generation ability of the PS-CDs was quantified using the DCFH-DA probe. Briefly, gradient concentrations of the PS-CDs solutions were incubated with 10 µM DCFH-DA for 30 min.^25^ Subsequently, the mixtures were exposed to ultrasound irradiation (1.0 MHz, 1.0 W cm^−2^, 6 min, 50% duty cycle) under dark conditions. To investigate the effect of ultrasound duration on ROS production, the PS-CDs solution (25 µg mL^−1^) was mixed with DCFH-DA and subjected to ultrasound irradiation for different time intervals ranging from 1 to 8 min. The fluorescence intensity of the oxidized DCF was then measured at an excitation wavelength of 488 nm and an emission range of 400–550 nm to quantify the ROS yield. The control groups consisting of the PS-CDs and DCFH-DA mixture without ultrasound activation were also prepared under the same conditions. Each sample was prepared in triplicate.

To identify the ROS species involved in the sonodynamic process, singlet oxygen (^1^O_2_) generation was evaluated using 1,3-diphenylisobenzofuran (DPBF) as a chemical probe. A fresh DPBF stock solution was prepared in ethanol and protected from light. The DPBF working solution was mixed with PS-CDs or PEG-CDs to obtain a final DPBF concentration of 20 µM. For the concentration-dependent assay, different concentrations of PS-CDs or PEG-CDs (0–100 µg mL^−1^) were mixed with DPBF and exposed to ultrasound irradiation at 1.0 MHz, 1.0 W cm^−2^, and a 50% duty cycle for 6 min. For the time-dependent assay, PS-CDs or PEG-CDs at 100 µg mL^−1^ were irradiated for 0–10 min under otherwise identical conditions. Corresponding samples without ultrasound irradiation were maintained in the dark for the same duration. The absorbance of DPBF at approximately 410 nm was measured using a microplate reader. Material blanks without DPBF were used to correct for the intrinsic absorbance and light scattering of the CDs.

Hydroxyl radical (˙OH) generation was assessed by monitoring the oxidative degradation of methylene blue (MB). An aqueous MB working solution was mixed with PS-CDs or P-CDs to obtain a final MB concentration of 25 µM. For the concentration-dependent assay, different concentrations of PS-CDs or PEG-CDs (0–180 µg mL^−1^) were mixed with MB and irradiated with ultrasound at 1.0 MHz, 1.0 W cm^−2^, and a 50% duty cycle for 6 min. For the time-dependent assay, PS-CDs or PEG-CDs at 180 µg mL^−1^ were irradiated for 0–10 min. Non-irradiated samples were incubated under the same conditions as controls. The absorbance of MB at approximately 664 nm was recorded using a microplate reader, with material blanks without MB used for background correction. All experiments were performed in triplicate and the results are expressed as mean ± SD.

### 2.5. Antioxidant capacity assay of CD

The total antioxidant capacity of silkworm excrement-derived PS-CDs was evaluated using the ABTS assay kit (E2006, APPLYGEN) for liquid samples.^26^ Briefly, equal volumes of ABTS (2,2′-azino-bis(3-ethylbenzothiazoline-6-sulfonic acid)) solution and oxidant were mixed under dark conditions and incubated for 6 h to generate the ABTS˙^+^ working solution. Subsequently, varying concentrations of PS-CDs solutions (0, 100, 300, 500, and 700 µg mL^−1^) were thoroughly mixed with the ABTS˙^+^ working solution and incubated for 25 min. The optical density was measured at 734 nm using a microplate reader (SpectraMax iD5, Molecular Devices), with the absorbance of PBS as the blank control (*A*_0_). Additionally, the 700 µg mL^−1^ PS-CDs solution was mixed with the ABTS˙^+^ working solution and incubated for different durations (0, 5, 10, 15, 20, and 25 min) to assess the time-dependent antioxidant capacity of the PS-CDs. The ABTS˙^+^ clearance rate (%) was calculated using the formula: (*A*_0_ − *A*_1_)/*A*_0_ × 100%, where *A*_1_ represents the absorbance of the sample and *A*_0_ represents the absorbance of the blank control.

To evaluate the free radical scavenging capability of the PS-CDs, the DPPH radical scavenging assay kit (AKAO020M, Boxbio) was employed.^27^ First, the DPPH working solution was prepared according to the kit instructions. Then, different concentrations of PS-CDs solutions (0, 100, 200, 300, 400, and 500 µg mL^−1^) were thoroughly mixed with the DPPH˙ working solution and incubated at room temperature for 25 min. The absorbance was measured at 515 nm using a multimode microplate reader, with PBS as the blank control (*A*_0_). The absorbance of the sample was denoted as *A*_1_. To assess the time-dependent antioxidant capacity, the 500 µg mL^−1^ PS-CDs solution was mixed with the DPPH˙ working solution and incubated for different durations (0, 5, 10, 15, 20, and 25 min). The DPPH˙ clearance rate (%) was calculated using the formula: (*A*_0_ − *A*_1_)/*A*_0_ × 100%.

The superoxide scavenging activities of PS-CDs were assessed by generating superoxide by a xanthine (Xan)/xanthine oxidase (XOD) reaction.^28^ Briefly, the reaction mixture was prepared by mixing different concentrations of PS-CDs solutions (0, 30, 60, 90, 120, and 150 µg mL^−1^) with nitroblue tetrazolium (NBT) working solution and a superoxide anion generator (xanthine/xanthine oxidase system). The mixture was incubated at 37 °C for 25 min under dark conditions. The absorbance was measured at 560 nm using a microplate reader (SpectraMax iD5, Molecular Devices), with PBS as the blank control. The absorbance of the blank control was denoted as *A*_0_, and the absorbance of the sample was denoted as *A*_1_. To assess the time-dependent SOD-like activity, the 150 µg mL^−1^ PS-CDs solution was mixed with the NBT working solution and superoxide anion generator, and incubated for different durations (0, 5, 10, 15, 20, and 25 min). The superoxide anion clearance rate (%) was calculated using the formula: (*A*_0_ − *A*_1_)/*A*_0_ × 100%.

Then, the contributions of silkworm excrement-derived components to the antioxidant activity were evaluated by comparing PS-CDs with PEG-CDs prepared without silkworm extract. Trolox was used as a positive antioxidant control. To avoid interference from the intrinsic absorbance of the CDs, corresponding material blanks without radical probes were measured and subtracted from each sample. For the ABTS˙^+^ scavenging assay, the ABTS working solution was prepared by mixing equal volumes of ABTS solution and oxidant, followed by incubation in the dark for 6 h. PS-CDs, PEG-CDs, or Trolox at 700 µg mL^−1^ was mixed with the ABTS˙^+^ working solution and incubated in the dark for 25 min. The absorbance at 734 nm was measured using a microplate reader. For the DPPH˙ scavenging assay, PS-CDs, PEG-CDs, or Trolox at 500 µg mL^−1^ was mixed with the DPPH working solution and incubated at room temperature in the dark for 25 min. The absorbance was measured at 515 nm. Superoxide anions were generated using a xanthine/xanthine oxidase system. PS-CDs, PEG-CDs, or Trolox at 150 µg mL^−1^ was mixed with nitroblue tetrazolium and the superoxide-generating reagents, followed by incubation at 37 °C in the dark for 25 min. The absorbance was measured at 560 nm. The radical-scavenging activity was calculated as follows: (*A*_0_ − *A*_1_)/*A*_0_ × 100%. All experiments were performed using three independent assay replicates.

### 2.6. *In vitro* bacterial uptake assay of PS-CDs

MDR *Pseudomonas aeruginosa* (*P. aeruginosa*) strain was kindly provided by the First Affiliated Hospital of Henan University of Chinese Medicine. The bacteria were cultured in lysogeny broth (LB) medium under shaking at 37 °C and harvested at the logarithmic growth phase prior to experiments. The bacterial concentration was determined by measuring the optical density at 600 nm using UV-vis spectroscopy. For the bacterial uptake assay, the PS-CDs (50 µg mL^−1^) were mixed with the bacterial suspension (final concentration of 10^6^ CFU mL^−1^) and incubated in the dark on a shaker at 37 °C and 300 rpm for different time intervals (0, 4, 8, 12, and 24 h). After incubation, the mixture was centrifuged to remove unbound PS-CDs, and the bacterial pellet was collected. The fluorescence intensity of the internalized PS-CDs was then measured at an excitation wavelength of 488 nm and an emission wavelength near 680 nm to quantify bacterial uptake. Meanwhile, the red fluorescence within the bacteria was visualized using a laser scanning confocal microscope (CLSM, Zeiss LSM900) with excitation at 488 nm and a red-emission collection window encompassing the CD emission near 680 nm.

The contribution of PBA-containing groups to bacterial association was further evaluated using S-CDs prepared without PBA-PEG-PBA and a competitive blocking assay. Log-phase MDR *Pseudomonas aeruginosa* was adjusted to 1 × 10^6^ CFU mL^−1^ and divided into three groups: S-CDs, PS-CDs, and PBA-PEG-PBA blocking followed by PS-CDs. For the S-CD and PS-CD groups, bacterial suspensions were incubated with the corresponding CDs at 50 µg mL^−1^ for 12 h at 37 °C with shaking at 300 rpm. For the competitive blocking group, bacteria were first incubated with free PBA-PEG-PBA at 50 µg mL^−1^ for 2 h to occupy accessible *cis*-diol-containing surface sites and were subsequently treated with PS-CDs at 50 µg mL^−1^ for 12 h under the same conditions. After incubation, the bacteria were collected by centrifugation and washed three times with phosphate-buffered saline to remove unbound materials. Bacteria-associated red fluorescence was observed using CLSM with excitation at 488 nm and a red-emission collection window encompassing the CD emission near 680 nm. All images were acquired using identical laser power, detector gain, exposure, and image-processing parameters. The background-corrected mean red fluorescence intensity was quantified using Image J and normalized to the mean value of the S-CD group. Three independent biological experiments were performed.

### 2.7. *In vitro* antibacterial activity assay of PS-CDs

The antibacterial activity of PS-CDs was evaluated against the MDR *Pseudomonas aeruginosa* strain was evaluated using the plate count method. The bacterial suspension was divided into the following groups: control (PBS), US irradiation alone, PS-CDs alone, and PS-CDs combined with US irradiation. Briefly, the bacterial suspension (final concentration of 10^6^ CFU mL^−1^) was evenly mixed with the material solution at a PS-CDs concentration of 50 µg mL^−1^. The mixtures were incubated in the dark on a shaker at 37 °C and 300 rpm for 12 h. Subsequently, the samples in the US irradiation groups were exposed to US (1.0 MHz, 1.0 W cm^−2^, 50% duty cycle) for 6 min. Then, aliquots from the suspensions were serially diluted, and 100 µL of each diluted bacterial solution was plated on LB agar plates. The number of bacterial colonies was recorded after overnight culture at 37 °C. Colony counts were captured and recorded using an automatic colony counter. Each sample was prepared in quintuplicate.

### 2.8. Live/dead bacterial staining assay

The bacteria treated under different conditions were collected and washed five times with saline. Subsequently, the bacterial suspensions were incubated with a mixture of SYTO 9 (excitation: 488 nm, emission: 498 nm) and propidium iodide (PI, excitation: 536 nm, emission: 617 nm) in the dark for 30 min at room temperature.^29^ After being washed three times with PBS, 10 µL of each sample was heat-fixed on a glass slide and observed using a CLSM (ZEISS LSM900).

### 2.9. Detection of ROS in bacteria

The ROS-generating capacity of the PS-CDs within bacteria was evaluated using a DCFH-DA assay kit.^30^ DCFH-DA (10 µM) was added to each bacterial suspension and incubated in the dark for 30 min. Subsequently, the groups requiring US treatment (*i.e.*, the US alone group and the PS-CDs + US group) were exposed to US at 1.0 MHz, 1.0 W cm^−2^, and 50% duty cycle for 6 min. After treatment, bacterial pellets were collected by centrifugation at 3000 rpm for 5 min and washed three times with PBS to remove any unreacted DCFH-DA. The fluorescence intensity of DCF, resulting from the oxidation of DCFH-DA, was detected using CLSM (ZEISS LSM900) to evaluate the level of ROS generated within the bacteria. The fluorescence intensity was analyzed using ImageJ software. Each sample was prepared in triplicate.

### 2.10. Morphology investigation of bacteria

The bacteria from different treatment groups were collected by centrifugation at 3000 rpm for 5 min and subsequently fixed with 2.5% glutaraldehyde solution for 4 h. A series of ethanol solutions with increasing concentrations (30%, 50%, 70%, 80%, 90%, and 100%) was used to dehydrate the bacteria, with each concentration applied for 15 min.^31^ Finally, the bacterial suspension was deposited onto a silicon wafer, and the morphology was characterized using scanning electron microscopy (SEM).

### 2.11. Quantification of bacterial DNA and protein leakage

After different treatments, the bacterial suspensions were further incubated at 37 °C for 4 h. The samples were then centrifuged, and the supernatants were collected. The extracellular DNA content was quantified by measuring the absorbance at 260 nm using a NanoDrop spectrophotometer. Using identically prepared and treated bacterial suspensions, the supernatant containing leaked proteins was collected after centrifugation at 3000 rpm for 5 min. Aliquots (50 µL) were mixed with 200 µL of BCA working reagent and incubated at 37 °C for 30 min. The absorbance at 562 nm was measured, and the leaked total protein concentration was quantified against a standard curve. Each sample was prepared in quintuplicate.

Protein leakage assessment: using identically prepared and treated bacterial suspensions, supernatant containing leaked proteins was collected post-centrifugation (3000 rpm, 5 min). Aliquots (50 µL) were mixed with 200 µL BCA working reagent and incubated at 37 °C for 30 min. Absorbance at 562 nm was measured, with total protein quantified against a standard curve. Each sample was prepared in quintuplicate.

### 2.12. Immunofluorescence staining of macrophage polarization

RAW264.7 murine macrophages, purchased from Servicebio Technology Co., Ltd (Wuhan, China), were cultured and maintained in Dulbecco's Modified Eagle Medium (DMEM) supplemented with 10% fetal bovine serum (FBS), which was obtained from Beisuo (Henan) Biotechnology Co., Ltd, Zhengzhou, China.^32^ For experiments, the cells were seeded into 24-well plates containing sterile glass coverslips at a density of 2 × 10^5^ cells per well and cultured overnight to allow adherence. The cells were divided into the following groups: control, LPS alone, and LPS + PS-CDs. The final concentration of LPS was 1 µg mL^−1^, and that of PS-CDs was 50 µg mL^−1^. After 24 h of co-incubation, the cells were fixed with 4% paraformaldehyde solution for 15 min and then blocked with a solution containing 1% bovine serum albumin (BSA). Primary antibodies, including APC anti-mouse CD86 and CoraLite® Plus 488 anti-mouse CD206, were applied and incubated overnight at 4 °C, followed by five washes with TBST. Then, the cells were incubated with secondary antibodies for 1 h at room temperature. Subsequently, the cells were co-incubated with DAPI for 5 min. After washing with PBS, the glass coverslips with adherent cells were removed and mounted using an antifade mounting medium. The fluorescence images were observed using a CLSM (ZEISS LSM900).

### 2.13. *In Vitro* ROS scavenging

DCFH-DA was used to detect the intracellular ROS level.^25^ Prior to intervention, cellular oxidative stress models were established by preconditioning the cells with LPS (1 µg mL^−1^) for 2 h under normal culture conditions (37 °C, 5% CO_2_), followed by continuous treatment with PS-CDs (50 µg mL^−1^) for 24 h. Then, DCFH-DA (10 µM) was added in the dark. After co-incubation for 30 min, the cells were washed with PBS to remove the unloaded DCFH-DA probe, and the intracellular ROS level was visualized using a CLSM (ZEISS LSM900). The fluorescence intensity was analyzed using ImageJ software. Each sample was prepared in triplicate.

### 2.14. *In Vitro* cytotoxicity assessment

The cytotoxicity of PS-CDs toward MLE-12 lung epithelial cells and human umbilical vein endothelial cells (HUVECs), both purchased from Servicebio Technology Co., Ltd (Wuhan, China), was evaluated using the Cell Counting Kit-8 (CCK-8) assay. Briefly, cells were seeded into 96-well plates at a density of 5 × 10^3^ cells per well and cultured overnight. The culture medium was then replaced with fresh medium containing PS-CDs at concentrations of 0, 25, 50, 75, or 100 µg mL^−1^. After incubation for 24 h, 10 µL of CCK-8 reagent was added to each well, followed by incubation at 37 °C for 2 h. The absorbance at 450 nm was measured using a microplate reader. Cell viability was calculated as follows:
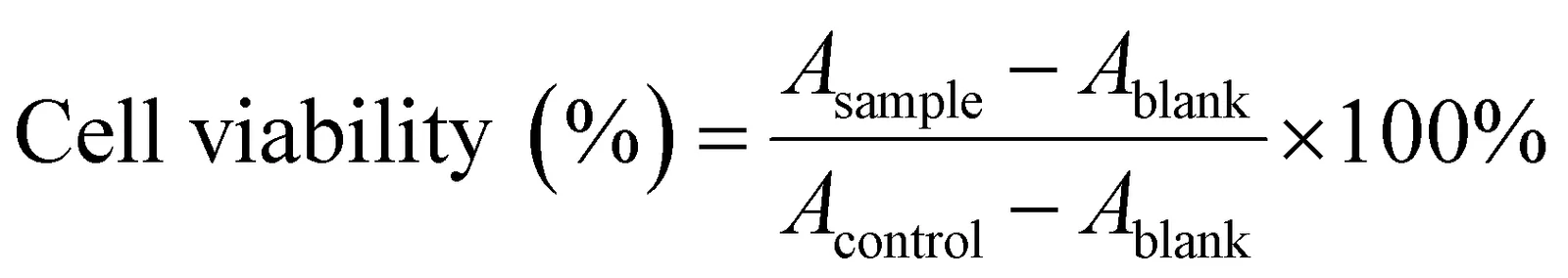
where *A*_sample_, *A*_control_, and *A*_blank_ represent the absorbance of PS-CD-treated cells, untreated cells, and cell-free wells containing culture medium and CCK-8 reagent, respectively.

### 2.15. Hemolysis assay

Blood samples were purchased from Beisuo (Henan) Biotechnology Co., Ltd (Zhengzhou, China), which collected them from healthy male BALB/c mice using heparinized tubes. The samples were centrifuged at 1500 rpm for 15 min to separate the plasma and buffy coat, which were then discarded to obtain packed red blood cells (RBCs). The RBCs were washed three times with sterile saline and resuspended in saline to prepare the RBCs stock solution (1 mL of packed RBCs in 4 mL of saline). Subsequently, 100 µL of the RBCs stock solution was mixed with 900 µL of PS-CDs at various concentrations (25, 50, 75, and 100 µg mL^−1^) and incubated at 37 °C for 4 h. After incubation, the mixtures were centrifuged at 3000 rpm for 10 min. Then, 200 µL of the supernatant from each sample was transferred to a 96-well plate, and the absorbance was measured at 540 nm using a microplate reader. Ultrapure water and saline were used as positive and negative controls, respectively. The hemolysis rate was calculated using the following formula:
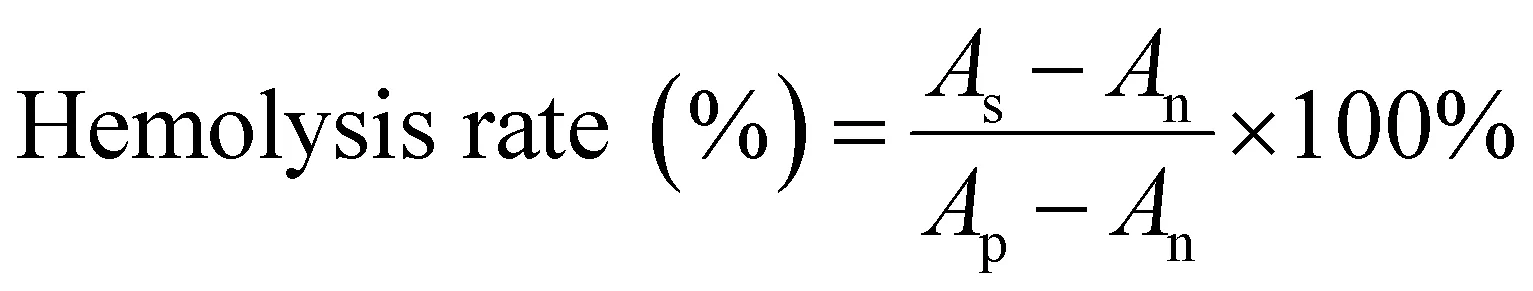
where *A*_s_, *A*_n_, and *A*_p_ represented the absorbance values of the test sample, negative control, and positive control, respectively.

### 2.16. Statistical analysis

GraphPad Prism software (version 9.0) and ImageJ software (version 2.1.0) were used for statistical analysis. All data were obtained from at least three parallel samples per condition in each experiment and are expressed as mean ± standard deviation (SD). All data differences were analyzed with one-way ANOVA. A probability value of *p* < 0.05 was considered statistically significant (*p < 0.05, **p < 0.01, ***p < 0.001).

## 3. Results and discussion

### 3.1. Preparation and characterization of the PS-CDs

**Fig. 2:**
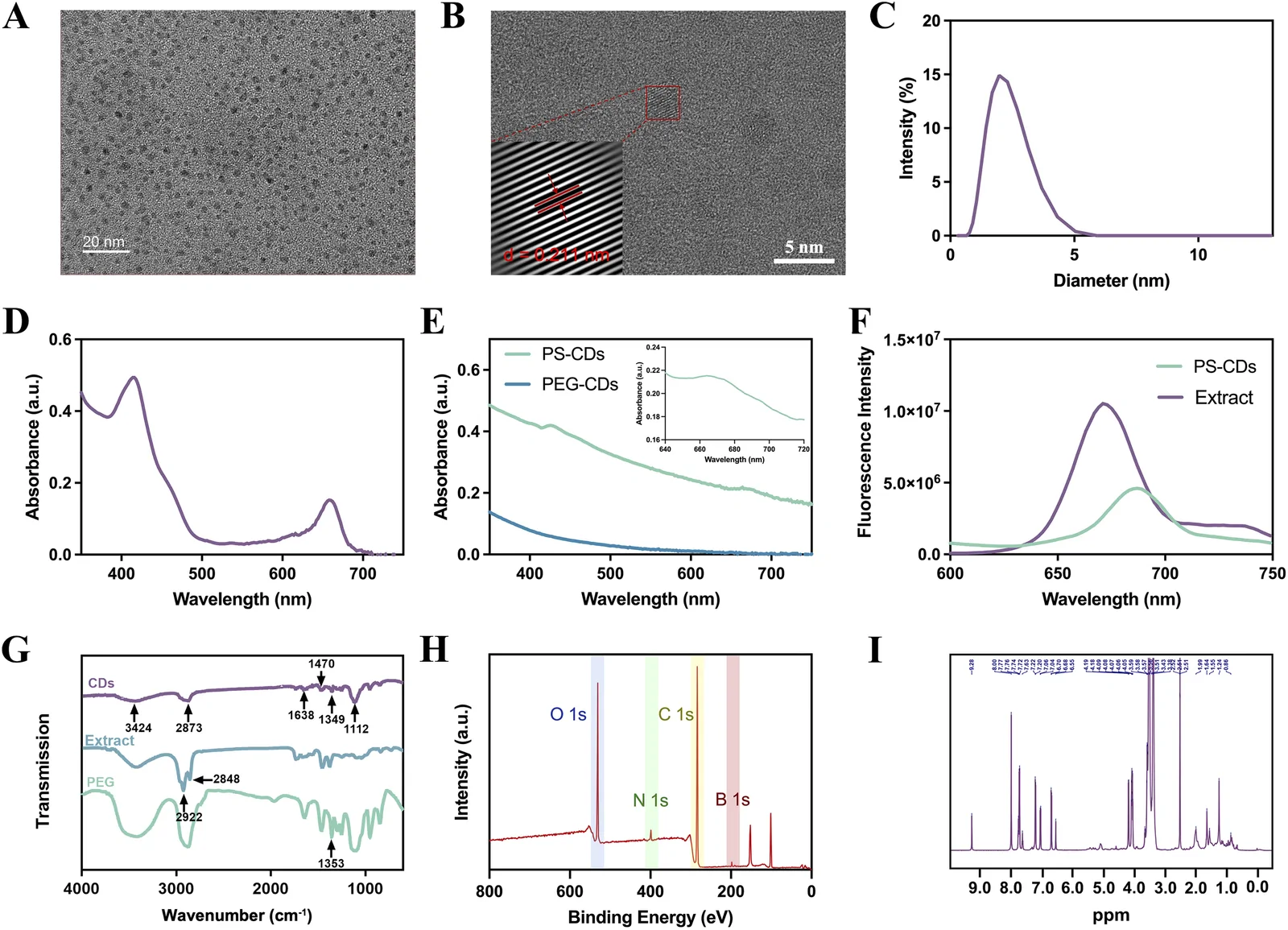
Characterization of PS-CDs. (A) TEM and (B) HRTEM images of PS-CDs. (C) Hydrodynamic size distribution of PS-CDs in deionized water. UV-vis-NIR absorption spectra of (D) silkworm excrement extract and (E) PEG-CDs and PS-CDs. (F) Fluorescence emission spectra of PS-CDs and silkworm excrement extract. (G) FTIR spectra of PS-CDs, silkworm excrement extract, and PBA-PEG-PBA. (H) XPS survey spectrum and (I) ^1^H NMR spectrum of PS-CDs.

Silkworm excrement-derived PS-CDs were prepared using a one-pot hydrothermal method. Showed uniformly dispersed, nearly spherical nanoparticles with diameters below 5 nm (Fig. 2A). High-resolution TEM revealed lattice fringes with a spacing of 0.211 nm (Fig. 2B), close to that of the (100) plane of graphitic carbon, indicating the formation of graphitic domains. Further XRD pattern showed that the PS-CDs exhibit a single, broad diffraction peak centered at approximately 2*θ* = 21° (Fig. S1). The center of this peak corresponds to an interlayer spacing of approximately 0.42 nm. Pristine graphite typically displays a tighter spacing of 0.34 nm. The shift of the peak to a lower angle and the resulting expanded interlayer spacing (0.42 nm) in our CDs is a well-documented phenomenon. This expansion is attributed to the introduction of abundant functional groups including hydroxyl, epoxy, and boric acids onto the surfaces and edges of the graphene-like planes during the synthesis process, which sterically pushes the layers apart. Dynamic light scattering (DLS) showed an average hydrodynamic diameter of approximately 3.8 nm and a polydispersity index (PDI) of 0.195 (Fig. 2C), suggesting a relatively narrow size distribution. The zeta potential was −0.54 mV, indicating a nearly neutral surface. This surface property may reflect the combined contributions of PEG chains, PBA-derived groups, and residual oxygen-containing groups on the PS-CDs.

The optical properties of PS-CDs were subsequently examined. Because of the large difference in absorbance intensity, the UV-vis-NIR spectra of the silkworm excrement extract and PS-CDs are presented separately in Fig. 2D and E. The extract exhibited pronounced absorption bands near 420 and 670 nm, corresponding to the Soret and Q-band regions of chlorophyll-related pigments, respectively.^22^ After hydrothermal carbonization, PS-CDs displayed a broad absorption profile with a weak shoulder near 670 nm rather than a well-resolved peak. Importantly, this feature was absent in PEG-CDs prepared from PBA-PEG-PBA alone, indicating that the absorption near 670 nm originates from silkworm excrement-derived components rather than from the PBA-PEG-PBA precursor. Together with the characteristic absorption of the original extract, these results support the partial retention of chlorophyll-derived chromophoric features in PS-CDs after hydrothermal treatment, although they do not indicate the preservation of intact chlorophyll molecules.

The fluorescence properties provided further information on the emissive states of PS-CDs. Upon excitation at 425 nm, PS-CDs exhibited red fluorescence with a maximum emission near 680 nm (Fig. 2F). A similar red-emission feature was observed for the silkworm excrement extract, further supporting the presence of chlorophyll-associated chromophoric characteristics in the carbonized product.^22^ The PLQY of PS-CDs was further determined to be 8.29% at an excitation wavelength of 678 nm, providing a quantitative measure of their red-emission efficiency. To investigate the excitation dependence of the photoluminescence, emission spectra were recorded at excitation wavelengths ranging from 638 to 688 nm (Fig. S2). Increasing the excitation wavelength progressively shifted the emission maximum from approximately 662 to 712 nm and altered the fluorescence intensity. This excitation-dependent photoluminescence is consistent with heterogeneous emissive states in CDs, which can arise from variations in surface functional groups, conjugated domains, and surface energy states.^33,34^ Thus, the optical data indicate that PS-CDs contain multiple emissive states together with red-emitting chromophoric features associated with the biomass precursor.

Fourier transform infrared (FTIR) spectroscopy was used to characterize the surface functional groups of PS-CDs. As shown in Fig. 2G, characteristic bands at approximately 3424, 2873, 1638, and 1112 cm^−1^ were assigned to O–H, C–H, C=O, and C–O–C vibrations, respectively. Additional bands at approximately 1470 and 1349 cm^−1^ were attributed to phenyl-ring skeletal and B–O vibrations. Together with the pronounced C–O–C ether band, these features support the incorporation of PBA-PEG-derived groups into PS-CDs. Compared with the silkworm excrement extract, changes in the C–H stretching region were also observed after hydrothermal treatment, indicating chemical transformation of the precursor components during CD formation.

X-ray photoelectron spectroscopy (XPS) provided complementary information on the elemental composition (Fig. 2H). The survey spectrum showed C 1s, N 1s, O 1s, and B 1s signals at approximately 284, 399, 531, and 198 eV, respectively. The C and O signals are consistent with the carbonaceous framework and oxygen-containing surface groups, while the N 1 s signal indicates the presence of nitrogen-containing species. Importantly, the B 1s signal (B content: 1.03 at%), together with the B–O and phenyl-ring vibrations detected by FTIR, provides complementary evidence for the incorporation of PBA-derived functionalities into PS-CDs. The ^1^H NMR spectrum further exhibited that multiple distinct peaks located in the region of *δ* 6.5–8.5 ppm are attributed to the aromatic protons attached to the sp^2^-hybridized carbon network, demonstrating the formation of a conjugated graphitic core alongside residual porphyrin-like macrocyclic fragments (Fig. 2I). Notably, the spectrum further showed a distinctive downfield resonance at approximately *δ* 9.28 ppm, consistent with strongly deshielded meso-protons within the chlorophyll-derived conjugated environments. When considered together with the weak absorption shoulder near 670 nm and red emission near 680 nm, these spectroscopic results support the presence of chlorophyll-derived chromophoric and tetrapyrrole-associated features after hydrothermal carbonization. Nevertheless, these data are interpreted as evidence for partial retention of precursor-derived chromophoric characteristics rather than preservation of intact chlorophyll molecules.

Finally, the colloidal stability of PS-CDs was evaluated in PBS, LB medium, and DMEM containing 10% FBS. Hydrodynamic diameters were monitored by DLS over 96 h (Fig. S3). No progressive increase in particle size was observed in any of the three media, and the measured hydrodynamic diameters remained below approximately 7 nm throughout the observation period. A slightly larger apparent size was observed in serum-containing DMEM, which may result from interactions with serum proteins. Overall, PS-CDs maintained a stable dispersion without evident time-dependent aggregation under the tested conditions.

### 3.2. ROS generation and scavenging of the PS-CDs

**Fig. 3:**
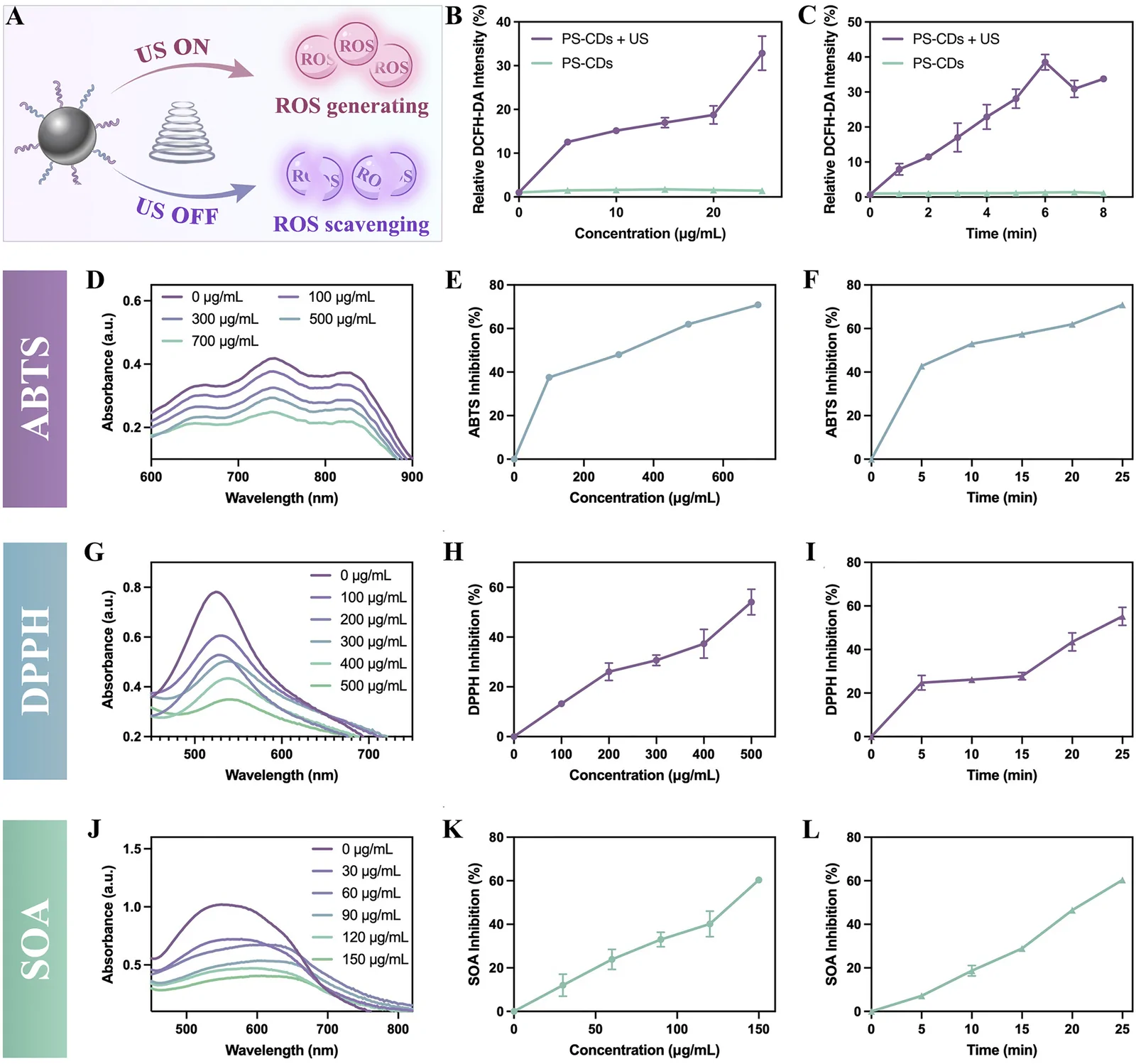
ROS generation and scavenging assay of the PS-CDs. (A) Schematic illustration of the ROS generation and scavenging processes by PS-CDs. (B and C) DCF fluorescence intensity after PS-CDs + US treatment as a function of PS-CDs concentration and irradiation time. (D–F) ABTS˙^+^ scavenging capacity of PS-CDs. (G–I) DPPH˙ scavenging capacity of PS-CDs. (J–L) Superoxide anion (SOA, O_2_˙^−^) scavenging capacity of PS-CDs.

Silkworm excrement contains chlorophyll-derived pigments, including pheophorbide a and purpurin 18, which have been reported to act as sonosensitizers and generate ROS under US irradiation.^25^ Given the chlorophyll-derived chromophoric features observed in PS-CDs, we first examined their US-triggered ROS generation using DCFH-DA as a general ROS indicator (Fig. 3B). Under US irradiation (1.0 MHz, 1.0 W cm^−2^, 50% duty cycle), DCF fluorescence increased with increasing PS-CD concentration, whereas only a weak signal was detected without US. ROS generation also increased with irradiation time and approached a plateau at approximately 6 min (Fig. 3C). These results confirm the US-dependent ROS-generating activity of PS-CDs and support the use of 6 min as an appropriate irradiation duration under the tested conditions. To further examine the ROS species involved, DPBF and MB were used as indicators of ^1^O_2_ and ˙OH, respectively. PEG-CDs prepared from PBA-PEG-PBA alone served as a precursor-derived control. As shown in Fig. S4, PEG-CDs caused only minor changes in DPBF and MB absorbance, regardless of US exposure, indicating limited sonodynamic activity. PS-CDs without US also resulted in little probe consumption. In contrast, US-irradiated PS-CDs produced pronounced concentration- and time-dependent decreases in both DPBF and MB absorbance. These results are consistent with the US-triggered generation of both ^1^O_2_ and ˙OH by PS-CDs. The weak response of PEG-CDs further indicates that the sonodynamic activity of PS-CDs is mainly associated with components derived from the silkworm excrement precursor, consistent with the chlorophyll-derived chromophoric features identified by spectroscopic characterization.

In addition to bacterial killing, regulation of excessive oxidative stress may be beneficial for limiting infection-associated inflammatory damage. Persistent bacterial infection is frequently accompanied by elevated endogenous ROS, which disrupt redox homeostasis and contribute to sustained inflammatory responses. We therefore evaluated the radical-scavenging activity of PS-CDs using three *in vitro* assays targeting ABTS˙^+^, DPPH˙, and superoxide anions (SOA, O_2_˙^−^). In the ABTS assay, the characteristic absorbance of ABTS˙^+^ at 734 nm progressively decreased with increasing PS-CD concentration (Fig. 3D and E). Approximately 70% of ABTS˙^+^ was scavenged by 700 µg mL^−1^ PS-CDs after 15 min. At this concentration, scavenging efficiency further increased with incubation time (Fig. 3F). Similar concentration- and time-dependent behavior was observed for DPPH˙ (Fig. 3G–I), with approximately 70% scavenging at 500 µg mL^−1^. PS-CDs also scavenged O_2_˙^−^ in a concentration- and time-dependent manner, reaching approximately 70% scavenging at 150 µg mL^−1^. Together, these assays demonstrate that PS-CDs can scavenge several chemically distinct radical species.

To determine the contribution of the silkworm excrement-derived components and benchmark the antioxidant activity, PEG-CDs and Trolox were evaluated under the same assay conditions (Fig. S5). PEG-CDs showed only limited scavenging of ABTS˙^+^, DPPH˙, and O_2_˙^−^, whereas PS-CDs exhibited substantially higher activity in all three assays. This comparison indicates that the radical-scavenging activity of PS-CDs is mainly associated with components derived from silkworm excrement. Compared with Trolox, PS-CDs showed lower ABTS˙^+^ and DPPH˙ scavenging activities but comparable O_2_˙^−^ scavenging activity. Thus, the antioxidant performance of PS-CDs varies among radical species and is not uniformly superior to that of a conventional small-molecule antioxidant. The relatively high concentrations used in these cell-free assays were intended to characterize the concentration-dependent scavenging capacity rather than to define a therapeutic concentration *in vivo*. Within the present platform, this intrinsic antioxidant activity provides a complementary function to US-activated ROS generation.

Importantly, the transition between ROS generation and scavenging is externally governed by the application of US rather than by an autonomous structural switch of PS-CDs. During the short period of US irradiation, sonodynamic activation favors transient ROS generation for bacterial killing. After US cessation, sonodynamic ROS production terminates, and the short-lived ROS generated during irradiation rapidly decay. The intrinsic radical-scavenging activity of PS-CDs, however, remains available to reduce excess ROS associated with persistent infection and inflammation. Thus, ROS generation is expected to dominate during US treatment, whereas radical scavenging can contribute during the subsequent post-treatment phase. This externally controlled sequence provides the mechanistic basis for the complementary ROS-generating and ROS-scavenging functions of PS-CDs.

### 3.3. *In vitro* antibacterial activity assay of PS-CDs

**Fig. 4:**
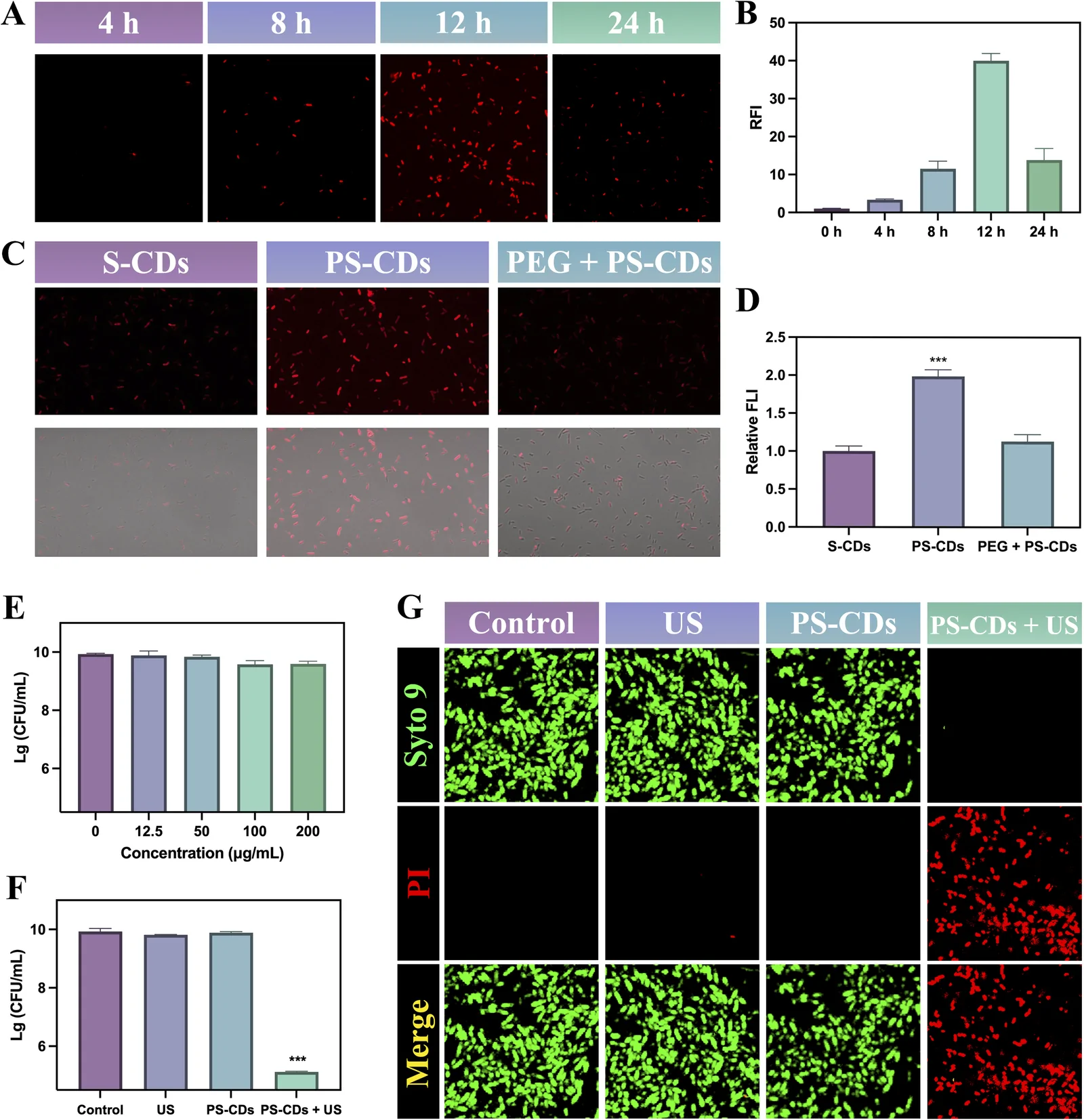
*In vitro* antibacterial effects of PS-CDs against MDR *P. aeruginosa*. (A) Representative CLSM images showing the time-dependent association of PS-CDs with MDR *P. aeruginosa*. (B) Semi-quantitative analysis of bacteria-associated PS-CD fluorescence. (C) Representative CLSM images of bacteria incubated with S-CDs, PS-CDs, or PS-CDs after 2 h of competitive blocking with free PBA-PEG-PBA. The upper row shows CD-derived red fluorescence, and the lower row shows the corresponding merged fluorescence and bright-field images. (D) Quantification of background-corrected bacteria-associated red fluorescence. ***p < 0.001 *versus* the S-CDs group. (E) Bacterial viability after treatment with varying concentrations of PS-CDs without US exposure. (F) Bacterial viability after treatment with PBS (control), US alone, PS-CDs alone, or PS-CDs + US. US parameters: 1.0 MHz, 1.0 W cm^−2^, and 50% duty cycle for 6 min. PS-CDs concentration: 50 µg mL^−1^. (G) Representative images of the Syto 9/PI-stained bacteria after various treatments.

*P. aeruginosa* is an important opportunistic pathogen frequently associated with difficult-to-treat hospital-acquired infections. A clinically isolated MDR *P. aeruginosa* strain was therefore selected as the primary Gram-negative model to evaluate the sonodynamic antibacterial activity of PS-CDs. The PBA-derived surface groups of PS-CDs can reversibly interact with *cis*-diol-containing structures on bacterial surface glycans, potentially promoting close association between PS-CDs and bacteria. Their ultrasmall size may further facilitate bacterial uptake and increase the local availability of PS-CDs for subsequent sonodynamic treatment. We first examined the interaction of PS-CDs with MDR *P. aeruginosa* after different incubation periods (Fig. 4A and B). Confocal laser scanning microscopy (CLSM) showed red fluorescence distributed throughout the bacterial profiles rather than isolated fluorescent spots. The bacteria-associated fluorescence progressively increased with incubation time and reached a maximum at approximately 12 h. These observations indicate efficient association of PS-CDs with bacteria and are consistent with bacterial uptake. When the incubation period was extended to 24 h, the fluorescence intensity decreased. This change may reflect dynamic interactions between PS-CDs and bacteria, or changes in fluorescence stability during prolonged incubation. Based on these results, 12 h was selected as the incubation period for subsequent antibacterial experiments.

To clarify the contribution of PBA-derived groups to bacterial association, S-CDs prepared from silkworm excrement extract without PBA-PEG-PBA were used as a non-PBA control. A competitive blocking experiment was also performed by preincubating bacteria with free PBA-PEG-PBA for 2 h before PS-CDs treatment. As shown in Fig. 4C and D, bacteria treated with S-CDs exhibited relatively weak and heterogeneous red fluorescence, with many bacteria showing little detectable signal. In contrast, PS-CDs treatment resulted in substantially stronger bacteria-associated fluorescence, approximately twice that observed with S-CDs. Preincubation with free PBA-PEG-PBA markedly reduced subsequent PS-CDs association, resulting in fluorescence close to that of the S-CDs group. The reduced bacterial association observed after either removal of the PBA-containing precursor or competitive blocking supports the contribution of PBA-mediated interactions with *cis*-diol-containing structures on the bacterial surface.

We next examined the antibacterial activity of PS-CDs in the absence and presence of US. PS-CDs alone caused no appreciable reduction in CFU at concentrations up to 200 µg mL^−1^ (Fig. 4E), indicating minimal intrinsic antibacterial activity without US activation. In contrast, combination with US substantially enhanced bacterial killing. At 50 µg mL^−1^, PS-CDs + US produced a 4.81 log_10_ reduction in viable MDR *P. aeruginosa*, whereas US alone showed little antibacterial effect (Fig. 4F). These results indicate that the antibacterial activity of PS-CDs is primarily dependent on US-mediated sonodynamic activation. Live/dead staining was further performed using SYTO 9 to label bacteria with intact membranes in green and PI to identify membrane-compromised bacteria in red. The control, US-only, and PS-CD-only groups exhibited predominantly green fluorescence (Fig. 4G). In contrast, the PS-CDs + US group showed strong red fluorescence, consistent with extensive membrane disruption following sonodynamic treatment.

To distinguish the contributions of the two precursor components, PEG-CDs, S-CDs, and PS-CDs were compared under identical US conditions. As shown in Fig. S6, PEG-CDs + US caused little change in viable bacterial counts compared with the control, consistent with the limited US-triggered ROS generation observed for PEG-CDs. In comparison, S-CDs + US reduced viable bacterial counts by approximately 2 log_10_, indicating that silkworm excrement-derived components contribute substantially to sonodynamic antibacterial activity. PS-CDs + US produced a greater reduction of 4.81 log_10_. Together with the bacterial association experiments, these findings suggest complementary contributions from the two precursors. The silkworm excrement-derived components provide the major contribution to US-triggered ROS generation, consistent with the presence of chlorophyll-associated chromophoric features, whereas PBA-derived surface groups enhance bacterial association and thereby increase the local availability of PS-CDs at the bacterial interface. The combination of these properties contributes to the greater sonodynamic antibacterial activity of PS-CDs.

The antibacterial activity of PS-CDs was further evaluated against methicillin-resistant *Staphylococcus aureus* (MRSA) as a representative Gram-positive MDR bacterium. Neither US nor PS-CDs alone substantially affected MRSA viability, whereas PS-CDs + US reduced viable bacterial counts by 2.64 log_10_ (Fig. S7). This reduction was lower than the 4.81 log_10_ reduction observed against MDR *P. aeruginosa*, indicating species-dependent antibacterial efficacy. Differences in bacterial envelope architecture and surface composition may affect PS-CD association, ROS accessibility, and susceptibility to oxidative damage. Overall, PS-CDs exhibited US-dependent antibacterial activity against both MDR *P. aeruginosa* and MRSA, although the magnitude of the effect differed between the two strains. Further studies involving additional bacterial species and clinical isolates will be required to define the antibacterial spectrum of PS-CDs more comprehensively.

### 3.4. *In vitro* antibacterial mechanism of PS-CDs

**Fig. 5:**
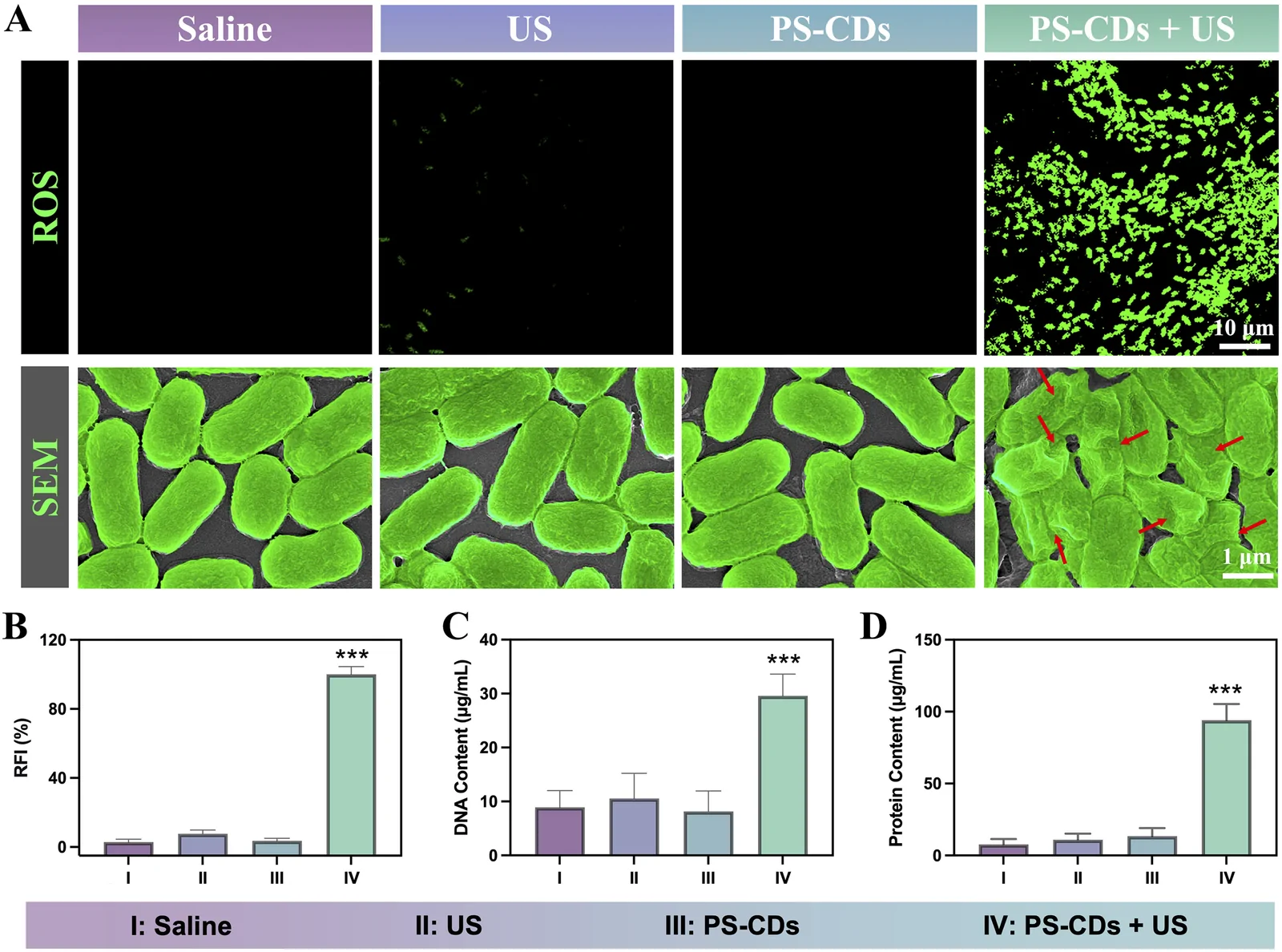
*In vitro* antibacterial mechanism of PS-CDs. (A) Representative CLSM images of DCF fluorescence and corresponding SEM images showing bacterial morphology after different treatments. (B) Semi-quantitative analysis of intracellular ROS levels based on DCF fluorescence. (C) DNA and (D) levels in bacterial supernatants after the indicated treatments.

ROS generation is an important mediator of sonodynamic antibacterial activity. We therefore examined intracellular ROS levels in MDR *P. aeruginosa* using DCFH-DA as a general ROS indicator. DCFH-DA can enter bacterial cells and is converted to non-fluorescent DCFH, which produces fluorescent DCF upon oxidation by ROS. As shown in Fig. 5A, bacteria in the PBS, US-only, and PS-CD-only groups exhibited weak green fluorescence. In contrast, the PS-CDs + US group showed markedly enhanced DCF fluorescence. Semi-quantitative analysis confirmed a significant increase in intracellular ROS after PS-CDs + US treatment (Fig. 5B). These results are consistent with the cell-free ROS measurements and indicate that US activation of bacteria-associated PS-CDs increases intracellular oxidative stress. The enhanced bacterial association mediated by PBA-derived groups may increase the local availability of PS-CDs at the bacterial interface, thereby facilitating ROS-mediated antibacterial activity upon US irradiation.

We next examined changes in bacterial morphology by SEM. Bacteria in the PBS, US-only, and PS-CD-only groups retained their typical rod-shaped morphology with relatively smooth and intact surfaces (Fig. 5A). In contrast, PS-CDs + US treatment resulted in pronounced surface wrinkling, contraction, and structural deformation, indicating substantial damage to the bacterial envelope. Consistent with these morphological changes, significantly higher levels of DNA and protein were detected in the supernatant of the PS-CDs + US group (Fig. 5C and D), indicating increased membrane permeability and leakage of intracellular components. Together, these findings suggest that US-activated PS-CDs increase intracellular oxidative stress and disrupt bacterial envelope integrity, leading to leakage of cellular components and loss of bacterial viability. These results provide mechanistic support for ROS-mediated membrane damage as an important contributor to the sonodynamic antibacterial activity of PS-CDs.

### 3.5. *In vitro* anti-inflammatory and redox-modulatory effects of PS-CDs

**Fig. 6:**
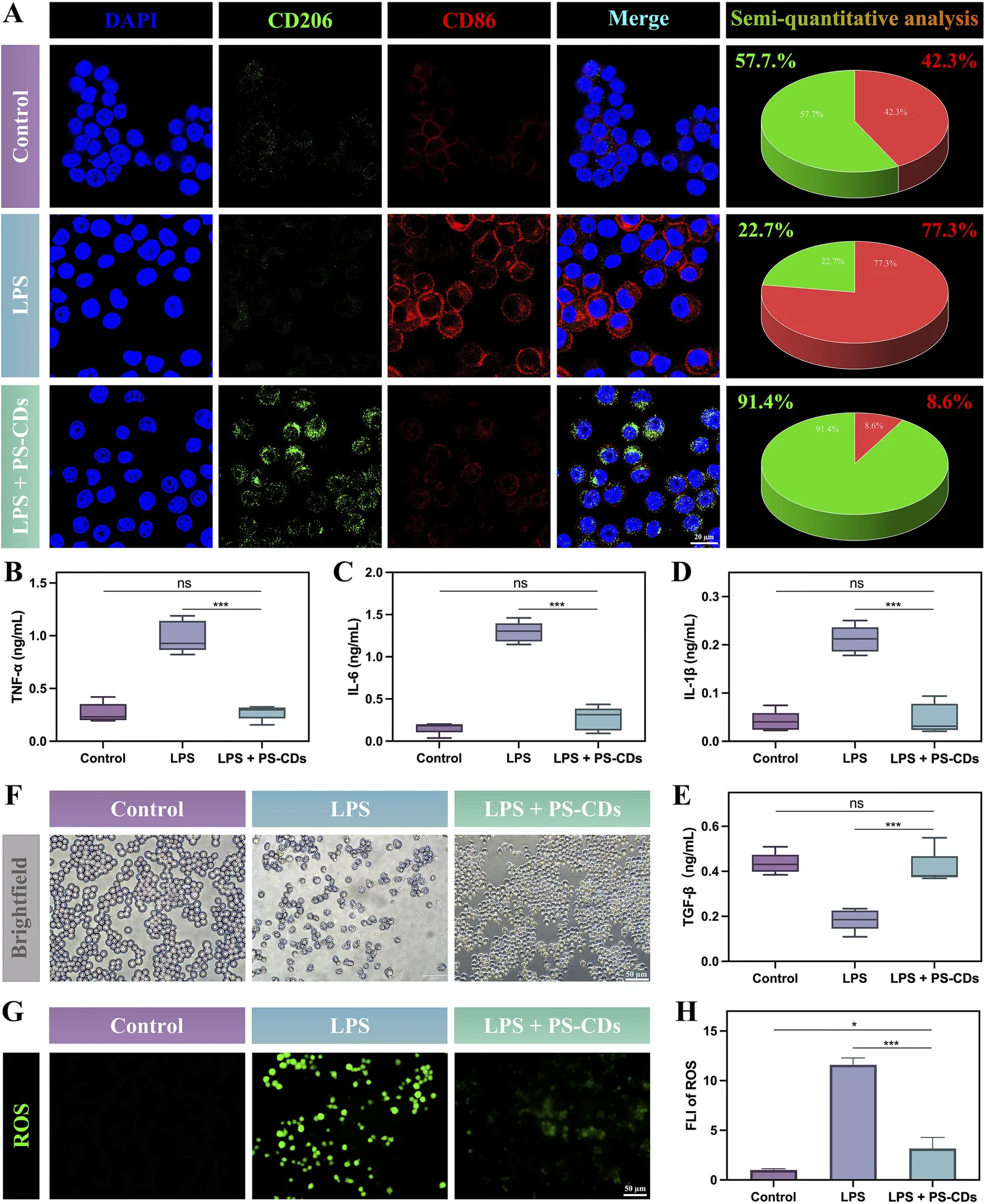
*In vitro* evaluation of the anti-inflammatory and redox-modulatory effects of PS-CDs. (A) Representative immunofluorescence images of CD86 (M1-associated marker, green) and CD206 (M2-associated marker, red) in RAW264.7 macrophages after the indicated treatments. (B–E) ELISA quantification of TNF-α, IL-1β, IL-6, and TGF-β secretion from RAW264.7 macrophages after different treatments. (F) Representative bright-field images and (G) CLSM images showing intracellular ROS in RAW264.7 macrophages. Green DCF fluorescence indicates intracellular ROS levels. (H) Semi-quantitative analysis of intracellular DCF fluorescence.

Macrophages play an important role in regulating inflammatory responses during bacterial infection. Prolonged inflammatory activation can increase ROS production and pro-inflammatory cytokine release, thereby contributing to sustained tissue injury. Given the radical-scavenging activity of PS-CDs, we investigated whether they could reduce oxidative stress and modulate the inflammatory phenotype of macrophages. RAW264.7 macrophages were stimulated with LPS (1 µg mL^−1^), and CD86 and CD206 were examined by immunofluorescence as M1- and M2-associated markers, respectively. As shown in Fig. 6A, unstimulated macrophages exhibited relatively weak fluorescence for both markers. LPS stimulation markedly increased CD86 fluorescence, consistent with pro-inflammatory activation. In comparison, treatment with PS-CDs reduced CD86 fluorescence and increased CD206 fluorescence. Semi-quantitative analysis showed that CD206-associated fluorescence accounted for 91.4% of the combined CD86/CD206 signal in the PS-CD-treated group. These results indicate a shift in macrophage marker expression toward a more anti-inflammatory phenotype. However, because this analysis is based on CD86/CD206 immunofluorescence, the 91.4% value represents the relative fluorescence contribution of CD206 rather than the percentage of cells undergoing M2 polarization. To further evaluate the inflammatory response, the culture supernatants were analyzed for TNF-α, IL-6, IL-1β, and TGF-β by enzyme-linked immunosorbent assay (ELISA) (Fig. 6B–E). Compared with LPS stimulation alone, PS-CD treatment partially reduced TNF-α, IL-6, and IL-1β secretion while increasing TGF-β secretion. These cytokine changes are consistent with the reduced CD86 and increased CD206 fluorescence and provide additional evidence that PS-CDs attenuate LPS-induced inflammatory activation.

We next examined macrophage morphology and intracellular ROS levels after different treatments. Bright-field imaging showed that untreated RAW264.7 macrophages maintained a relatively round and smooth morphology (Fig. 6F). Following LPS stimulation, the cells displayed pronounced morphological alterations, including cell shrinkage, irregular contours, and bleb-like surface changes. In comparison, macrophages treated with PS-CDs in the presence of LPS showed less pronounced morphological alterations and largely retained a rounded appearance. Intracellular ROS levels were subsequently evaluated using DCFH-DA (Fig. 6G). Unstimulated macrophages exhibited weak DCF fluorescence, whereas LPS stimulation markedly increased the intracellular fluorescence signal, indicating elevated oxidative stress. PS-CD treatment substantially reduced DCF fluorescence in LPS-stimulated macrophages, and semi-quantitative analysis confirmed this reduction (Fig. 6H). Together, the morphological observations, intracellular ROS measurements, macrophage-marker expression, and cytokine profiles indicate that PS-CDs attenuate LPS-induced oxidative and inflammatory responses *in vitro*. This antioxidant and inflammatory–modulatory activity provides a complementary function to the ultrasound-activated antibacterial activity of PS-CDs.

### 3.6. *In vitro* safety evaluation of PS-CDs

**Fig. 7:**
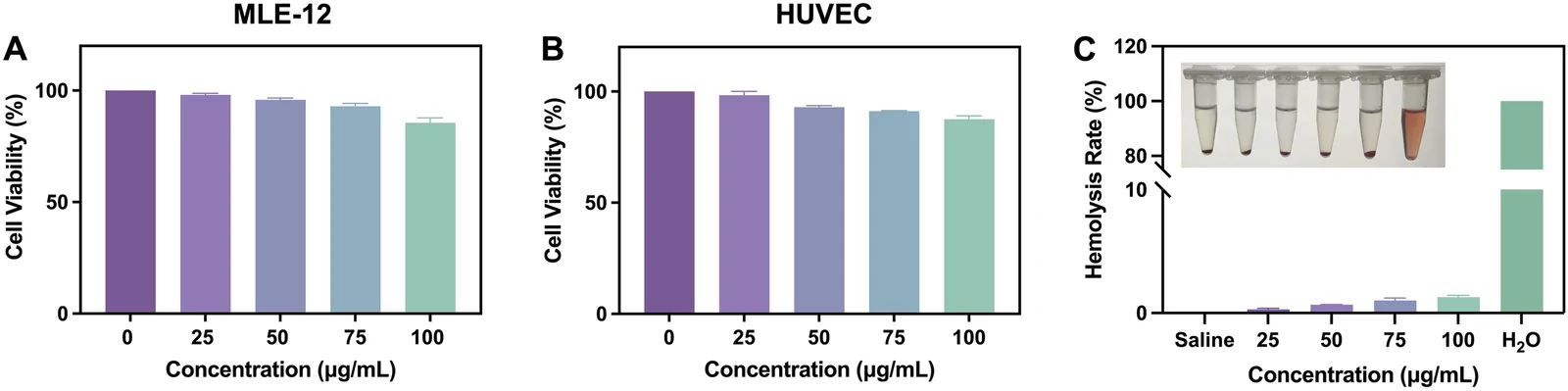
*In vitro* biocompatibility of PS-CDs. Viability of (A) MLE-12 cells and (B) HUVECs after incubation with PS-CDs (0–100 µg mL^−1^) for 24 h, as determined by the CCK-8 assay. (C) Hemolysis of mouse erythrocytes after incubation with different concentrations of PS-CDs at 37 °C for 4 h. Water and saline were used as positive and negative controls, respectively.

Biocompatibility is an important consideration for the further biomedical application of nanomaterials. We therefore evaluated the cytocompatibility of PS-CDs using MLE-12 lung epithelial cells and human umbilical vein endothelial cells (HUVECs). Cells were incubated with PS-CDs at concentrations ranging from 25 to 100 µg mL^−1^ for 24 h, followed by CCK-8 analysis. As shown in Fig. 7A and B, the viability of both cell types remained above 95% throughout the tested concentration range. No appreciable reduction in cell viability was observed even at 100 µg mL^−1^, which was twice the PS-CD concentration used for sonodynamic antibacterial treatment. These results indicate low cytotoxicity of PS-CDs under the tested conditions. Hemocompatibility was further evaluated using an erythrocyte hemolysis assay. Saline produced negligible hemolysis, whereas water caused extensive hemolysis and served as the positive control. PS-CDs exhibited hemolysis rates below 5% at concentrations of 25, 50, 75, and 100 µg mL^−1^ (Fig. 7C), indicating low hemolytic activity within the tested concentration range. Together, these results demonstrate favorable *in vitro* cytocompatibility and low hemolytic activity of PS-CDs, supporting their further evaluation as an antibacterial nanomaterial.

## 4. Conclusion

In summary, PBA-containing carbon dots (PS-CDs) were prepared from silkworm excrement extract and PBA-PEG-PBA through a one-pot hydrothermal process. Spectroscopic characterization supported the partial retention of chlorophyll-derived chromophoric and tetrapyrrole-associated features, while PBA-derived surface groups enhanced bacterial association. Under ultrasound irradiation, PS-CDs generated ROS, including ^1^O_2_ and ˙OH, and produced a 4.81-log_10_ reduction in viable MDR *P. aeruginosa*. Antibacterial activity was also observed against MRSA, although with a lower 2.64-log_10_ reduction, indicating species-dependent efficacy. In the absence of ultrasound, PS-CDs scavenged ABTS˙^+^, DPPH˙, and O_2_˙^−^ radicals, providing a complementary antioxidant function. In LPS-stimulated RAW264.7 macrophages, PS-CDs reduced intracellular ROS, shifted CD86/CD206 expression toward a less pro-inflammatory phenotype, partially decreased TNF-α, IL-6, and IL-1β secretion, and increased TGF-β secretion. PS-CDs also showed favorable *in vitro* cytocompatibility and low hemolytic activity. Together, these findings provide an *in vitro* proof of concept for integrating externally controlled sonodynamic bacterial killing with antioxidant and inflammatory modulation in a biomass-derived CD platform. Further studies in additional bacterial strains and appropriate animal infection models are required to evaluate its *in vivo* efficacy, biodistribution, and long-term safety.

## Author contributions

Yuxuan Ren: investigation, methodology, writing – original draft. Wenhui Cao: validation, investigation, methodology. Hui Wang: visualization, validation, investigation, formal analysis. Jianing Zheng: visualization, investigation, data curation. Yongfang Wang: investigation, methodology. Anqi Liu: investigation. Chao Liu: investigation. Xin Pang: conceptualization, funding acquisition, supervision, writing – review & editing. Zhenzhen Wang: conceptualization, supervision, writing – original draft.

## Conflicts of interest

The authors declare that they have no known competing financial interests or personal relationships that could have appeared to influence the work reported in this paper.

## Supplementary Material

RA-016-D6RA05433A-s001

## Data Availability

The data that support the findings of this study are available from the corresponding authors upon reasonable request. Supplementary information (SI) is available. See DOI: https://doi.org/10.1039/d6ra05433a.
