# Supplementary material for: Silkworm excrement-derived carbon dots for sonodynamic bacterial killing and antioxidant–inflammatory modulation
Source: RSC Adv. 2026 Sep 2;16(42):51704–18. doi: 10.1039/d6ra05433a (PMC13537384; doi:10.1039/d6ra05433a)
Supplement: Supplementary file 1 [file RA-016-D6RA05433A-s001.pdf]

Supplementary Information (SI) for RSC Advances.
This journal is © The Royal Society of Chemistry 2026

Supporting Information

Silkworm Excrement-Derived Carbon Dots for Sonodynamic Bacterial Killing and Antioxidant–Inflammatory Modulation

Yuxuan Ren[a, b, 1], Wenhui Cao[c, 1], Hui Wang[c], Jianing Zheng[c], Yongfang Wang[c], Anqi Liu[c], Chao Liu[c], Xin Pang[c, *], Zhenzhen Wang[a, *]

[a] Academy of Chinese Medical Sciences, Henan University of Chinese Medicine, Zhengzhou 450046, China.

[b] Department of Rheumatology, The First Affiliated Hospital of Henan University of Chinese Medicine, Zhengzhou 450046, China.

[c] School of Pharmacy, Henan University of Chinese Medicine, Zhengzhou 450046, China.

[1] These authors contributed equally to this work.

*Correspondence authors

E-mail: pangxin116@163.com (X. Pang), wzlinxi@163.com (Z.Z. Wang)

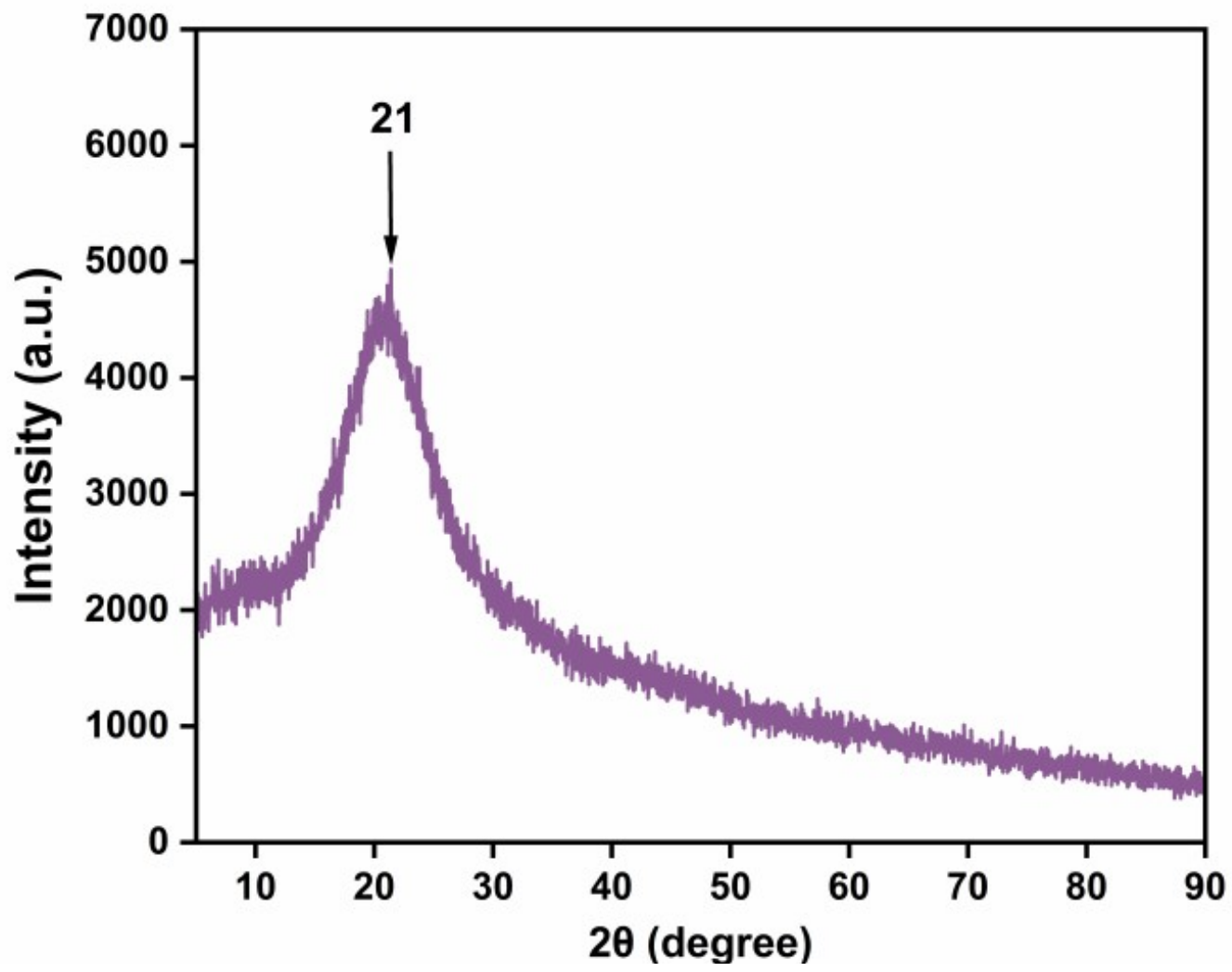

Fig. S1 XRD pattern of PS-CDs.

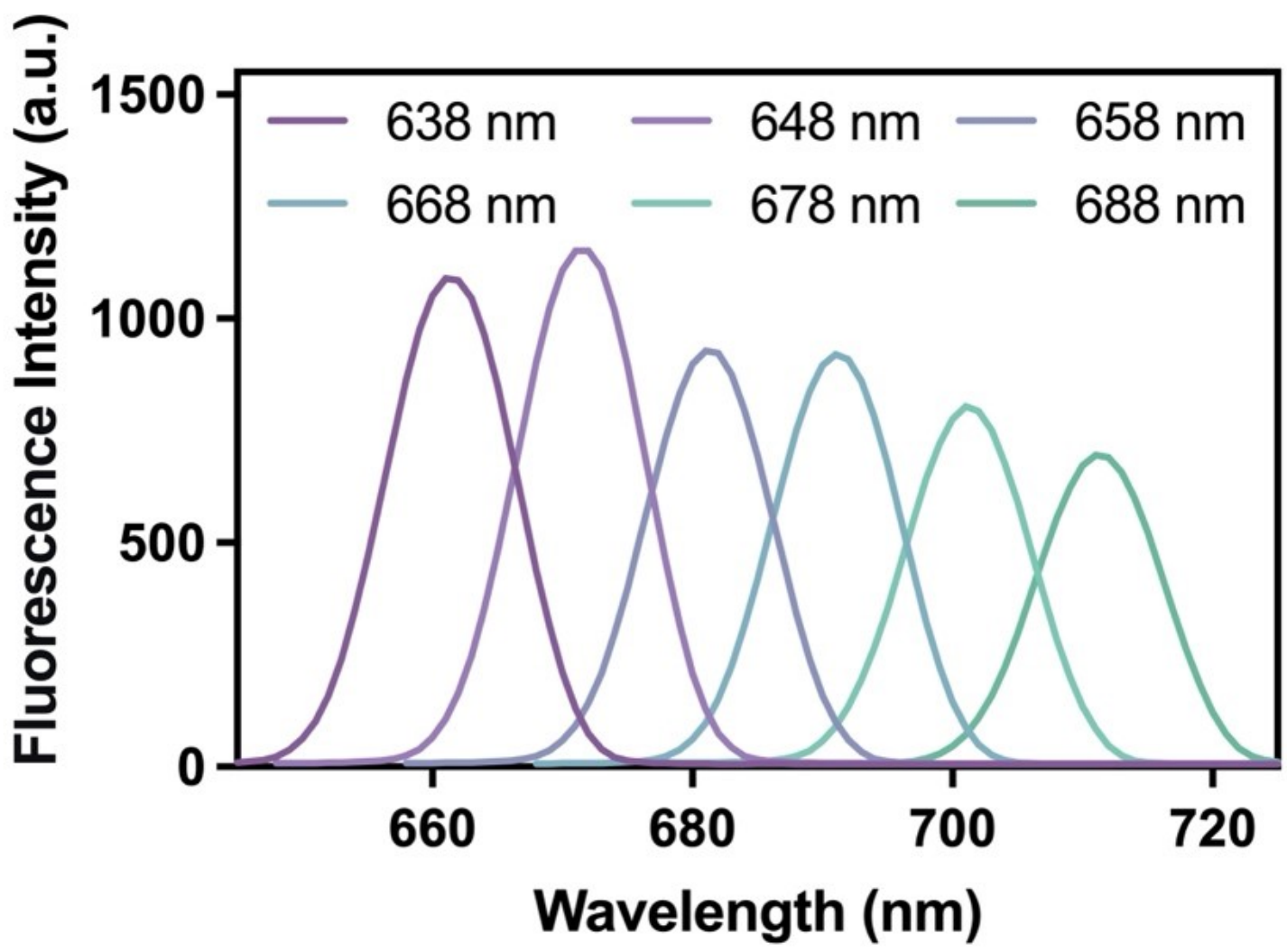

Fig. S2 Photoluminescence emission spectra of PS-CDs recorded at different excitation wavelengths (638–688 nm), showing excitation-dependent emission with a progressive red shift of the emission maximum.

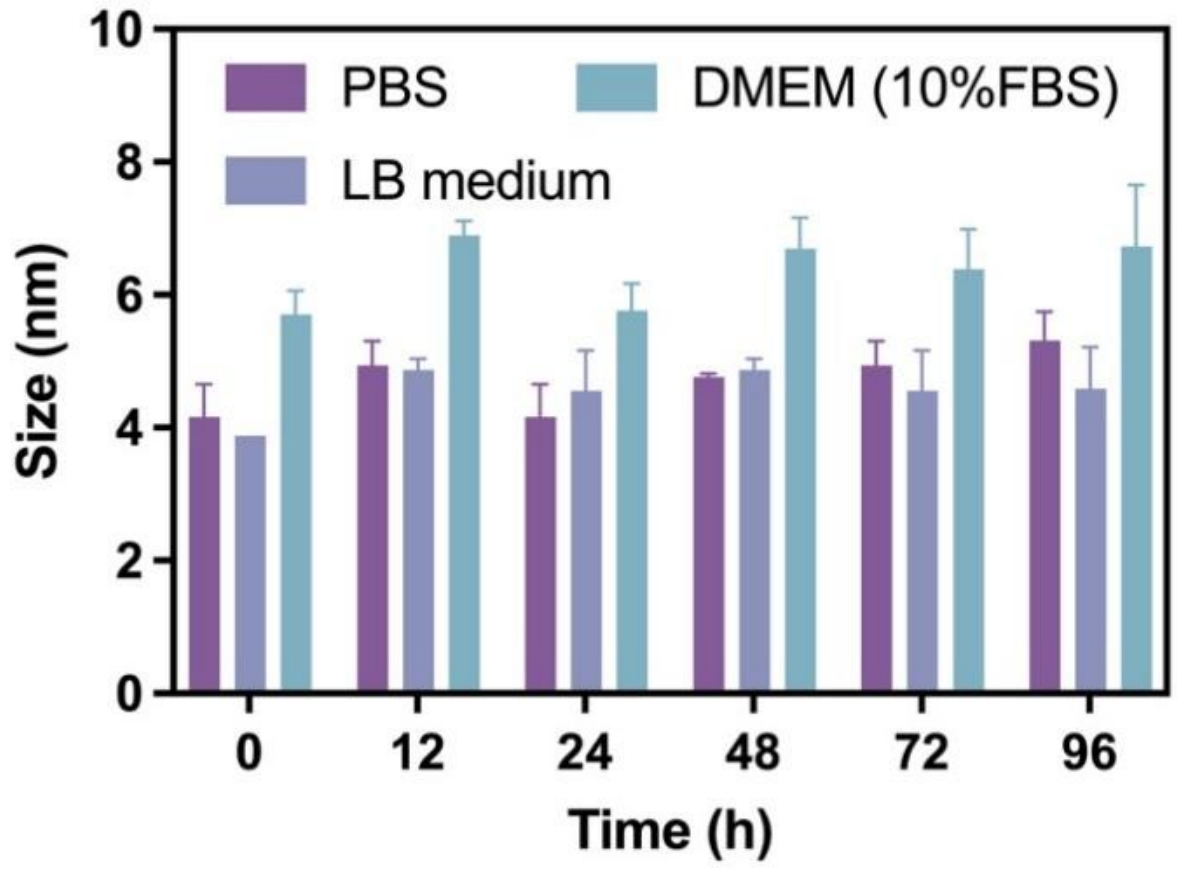

Fig. S3 Colloidal stability of PS-CDs in PBS, LB medium, and DMEM supplemented with 10% fetal bovine serum (FBS) was monitored by dynamic light scattering at 0, 12, 24, 48, 72, and 96 h.

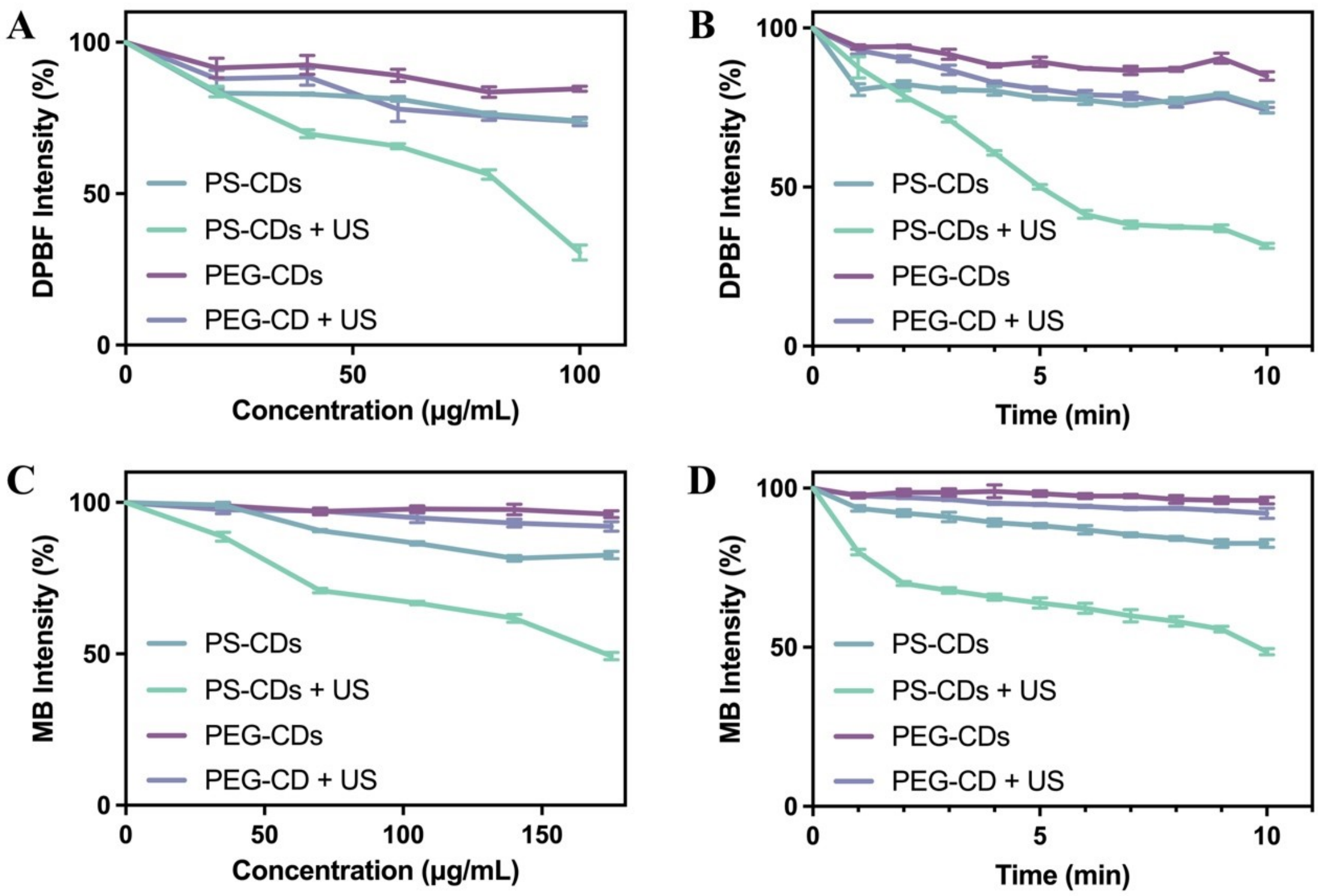

Fig. S4 Identification of ROS species generated by PS-CDs under ultrasound irradiation. (A, B) Concentration- and irradiation time-dependent consumption of 1,3-diphenylisobenzofuran (DPBF), indicating singlet oxygen ($^1O_2$) generation. (C, D) Concentration- and irradiation time-dependent degradation of methylene blue (MB), indicating hydroxyl radical (•OH) formation.

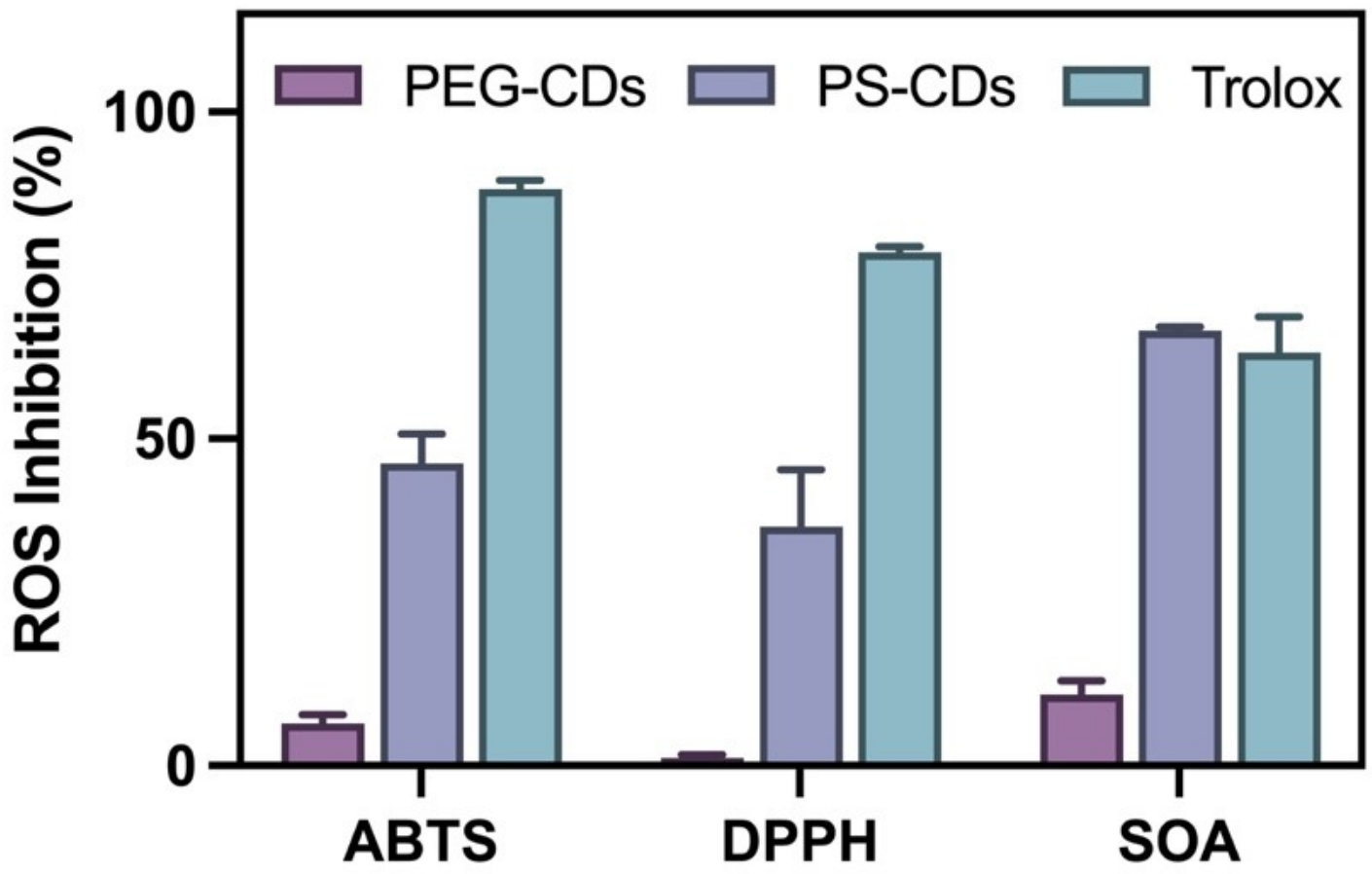

Fig. S5 ROS scavenging capacity of PEG-CDs, PS-CDs, and Trolox. Data are presented as mean ± SD (n = 3 independent assays).

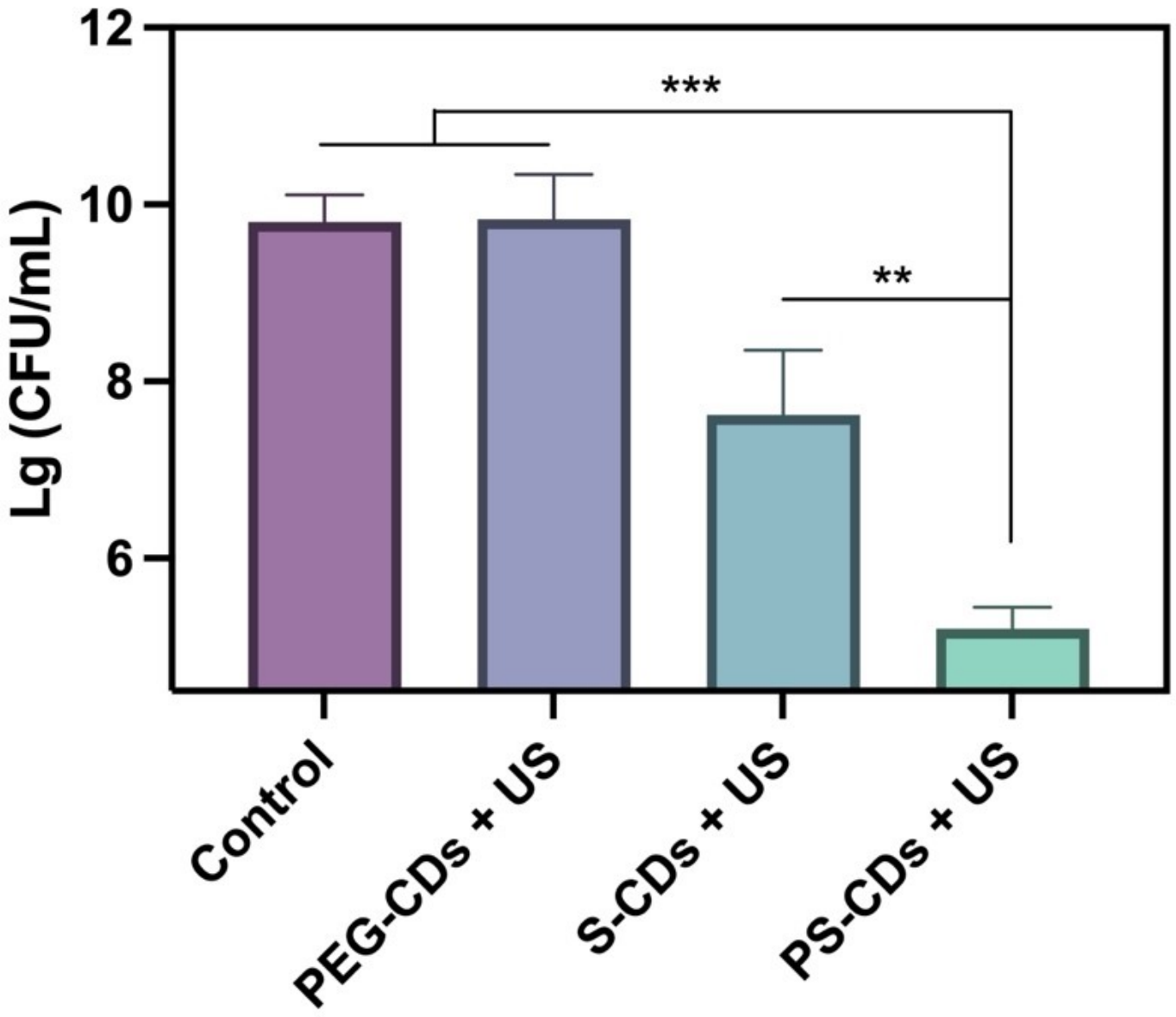

Fig. S6 Comparison of the ultrasound-mediated antibacterial activities of different CDs against MDR *Pseudomonas aeruginosa*. Bacteria were treated with PBS (control), PEG-CDs, S-CDs, or PS-CDs at 50 μg/mL, followed by ultrasound irradiation at 1.0 MHz, 1.0 W/cm^2, and a 50% duty cycle for 6 min. Viable bacterial counts were determined using the serial dilution plate-counting method and are expressed as $\log_{10}$ CFU/mL.

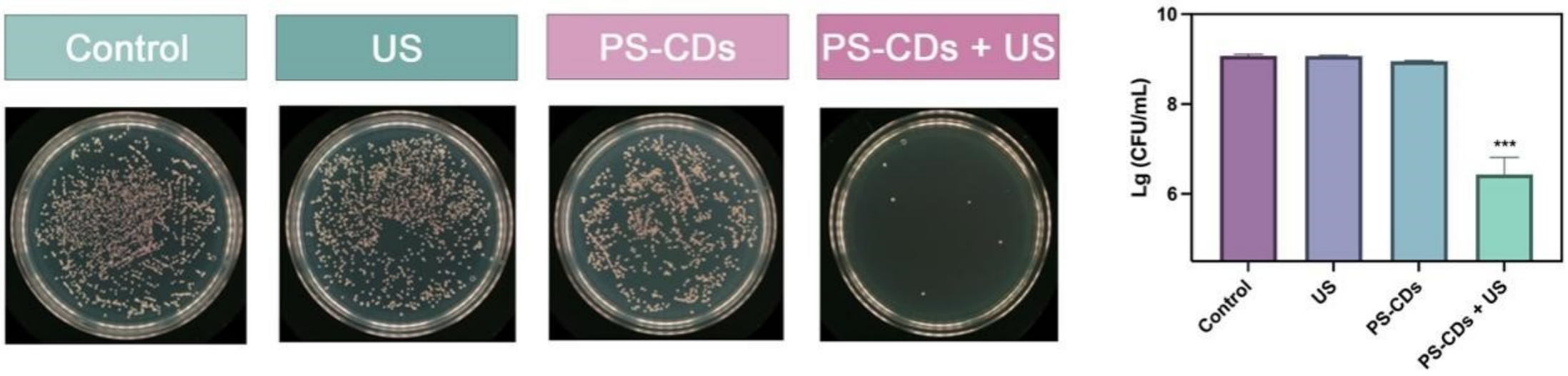

Fig. S7 *In vitro* antibacterial activity of PS-CDs against methicillin-resistant *Staphylococcus aureus* (MRSA). Representative agar-plate images and quantification of viable bacterial counts of MRSA after treatment with saline (control), US, PS-CDs, or PS-CDs + US. The PS-CD concentration was 50 μg/mL, and the US parameters were 1.0 MHz, 1.0 W/cm^2, a 50% duty cycle, and 6 min.
